# Development of a risk prediction model for dialysis access steal syndrome: exploring the interaction and modifying role of BMI

**DOI:** 10.3389/fpubh.2026.1790958

**Published:** 2026-04-07

**Authors:** Yan Liu, Zhenxia Huo, Xiaoran Gao, Xinyue Wang

**Affiliations:** 1 Department of Nephrology, The Second Affiliated Hospital of Xingtai Medical College, Xingtai, China; 2 Department of Nephrology (Traditional Chinese Medicine), The Second Affiliated Hospital of Xingtai Medical College, Xingtai, China; 3 Hemodialysis Unit, Department of Nephrology, The Second Affiliated Hospital of Xingtai Medical College, Xingtai, China

**Keywords:** dialysis access steal syndrome, effect modification, end-stage renal disease, frailty, interaction, prediction model, SHAP analysis

## Abstract

**Background:**

Dialysis access steal syndrome (DASS) is a severe complication of vascular access surgery. This study aimed to identify key risk factors and develop an interpretable prediction model for early risk assessment in patients with end-stage renal disease (ESRD).

**Methods:**

This retrospective study analyzed 324 ESRD patients (March 2023–June 2025). Feature selection was performed using LASSO regression combined with SHapley Additive exPlanations (SHAP). Independent risk factors were identified via multivariable logistic regression, with robustness confirmed by sensitivity analysis and E-values. Restricted cubic splines (RCS) explored non-linear associations, while BMI-stratified and interaction analyses evaluated effect modification. Model performance was validated using AUC, 1000-sample bootstrapping, and 10-fold cross-validation.

**Results:**

DASS occurred in 86 patients (26.5%). The Fried Frailty Score (FFS) emerged as the most significant contributor. Multivariable analysis identified FFS, BMI, CKD duration, sarcopenia, distal arterial pressure (DAP), and surgical experience as independent predictors. RCS analysis identified critical thresholds: BMI 23.3 kg/m^2^, DAP 66 mmHg, and CKD duration 8.2 years. Notably, BMI exerted significant effect modification (*P*_interaction_ < 0.05); FFS, sarcopenia, DAP, and CKD duration were significantly associated with DASS only in the low/normal BMI group. The final model demonstrated excellent discrimination (AUC = 0.934) and stability (Brier score = 0.085; C-index = 0.931). High-risk patients showed a significantly higher DASS incidence than low-risk patients (50.00% vs. 3.09%, *p* < 0.001).

**Conclusion:**

FFS, sarcopenia, BMI, DAP, and CKD duration are core predictors of DASS. BMI acts as a key effect modifier, particularly influencing the impact of functional and hemodynamic indicators. This high-performance model provides a scientific basis for personalized preoperative screening and clinical intervention.

## 1 Introduction

Vascular access is widely regarded as the “lifeline” for patients with end-stage renal disease (ESRD) undergoing hemodialysis (1). While the arteriovenous fistula (AVF) and arteriovenous graft (AVG) remain the gold standards for long-term access, they are not without significant complications (2). Among these, Dialysis Access Steal Syndrome (DASS)—or hemodialysis access-induced distal ischemia—is one of the most debilitating (3, 4). DASS occurs when the low-resistance vascular access “steals” arterial blood flow from the distal extremity, leading to symptoms ranging from coldness and paresthesia to digital ulceration, gangrene, and permanent neurological deficit (5, 6).

Despite advancements in surgical techniques, the incidence of symptomatic DASS remains a persistent challenge, particularly as the hemodialysis population ages and presents with more complex comorbidities (7). The pathophysiology of DASS is multifactorial, involving a delicate imbalance between access flow, systemic arterial pressure, and the compensatory capacity of the distal collateral circulation. Current clinical practice often relies on late-stage physical findings, yet irreversible ischemic damage can occur rapidly, highlighting an urgent need for early and precise risk stratification (8, 9).

Recent literature has identified several potential contributors to DASS, including diabetes mellitus, peripheral artery disease and specific anatomical configurations (10). However, existing prediction models often lack the integration of hemodynamic indicators and functional variables, and many fail to account for the non-linear biological relationships inherent in vascular pathology (11, 12). Furthermore, the role of Body Mass Index (BMI) as a potential effect modifier remains insufficiently explored, despite its known influence on both systemic cardiovascular resistance and surgical outcomes (13, 14).

To address these gaps, this study utilizes a robust machine-learning-enhanced framework. By combining least absolute shrinkage and selection operator (LASSO) regression for high-dimensional feature selection with SHapley Additive exPlanations (SHAP), we aim to provide a highly interpretable model that goes beyond simple “black-box” predictions. We further employ Restricted Cubic Splines (RCS) to elucidate non-linear associations and conduct interaction analyses to determine how BMI stratifies DASS risk.

By integrating clinical, functional, and hemodynamic indicators, this study seeks to construct a validated, clinician-friendly prediction tool to facilitate early intervention and improve the long-term limb prognosis for ESRD patients.

## 2 Materials and methods

### 2.1 Study flow and design overview

**Figure 1:**
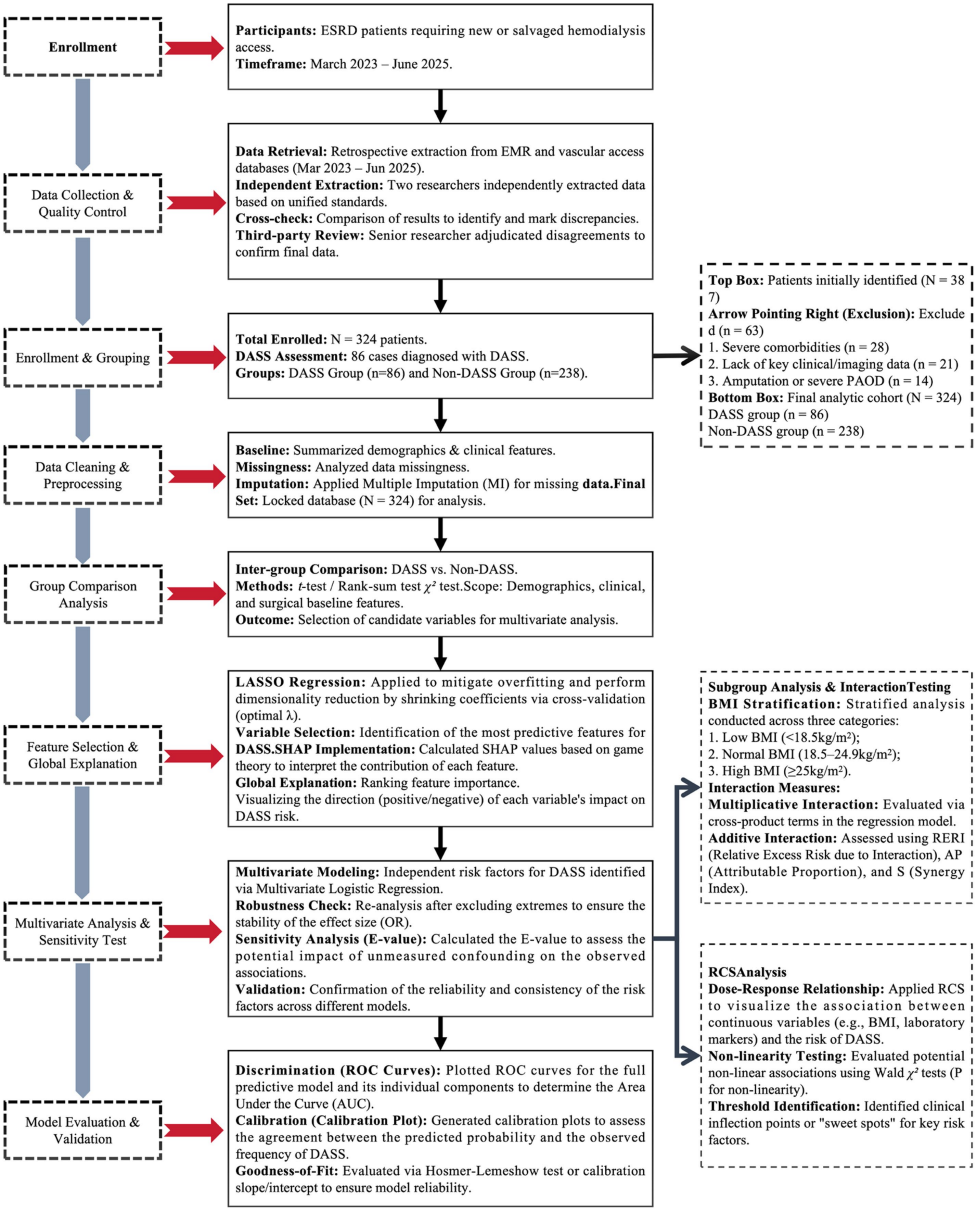
Overall study design flowchart.

This study followed a structured pipeline from participant enrollment to model validation (Figure 1).

1. *Enrollment and preprocessing*: Between March 2023 and June 2025, 324 ESRD patients were retrospectively enrolled and categorized into DASS (*n* = 86) and Non-DASS (*n* = 238) groups. Data underwent double-extraction with third-party adjudication, and missing values were addressed using Multiple Imputation to ensure a robust final dataset (*N* = 324).
2. *Feature selection and modeling*: After initial inter-group comparisons, LASSO regression was utilized for dimensionality reduction and variable selection via cross-validation. The predictive contribution of each feature was interpreted using SHAP values. Independent risk factors were then identified through Multivariate Logistic Regression, with model stability further confirmed via E-value sensitivity analysis and robustness checks.
3. *Advanced analysis and validation*: Restricted Cubic Splines (RCS) were employed to visualize non-linear dose–response relationships and identify clinical thresholds. Additionally, interaction testing (additive and multiplicative) was performed across BMI strata. Model performance was rigorously evaluated using ROC curves for discrimination and Calibration Plots (with Hosmer-Lemeshow tests) to ensure predictive accuracy.

### 2.2 Study design and participants

This study is a retrospective observational study conducted at the Second Affiliated Hospital of Xingtai Medical College. We identified and screened patients with ESRD who underwent vascular access surgery between March 2023 and June 2025. All patients received standardized post-operative follow-up to monitor vascular access function and assess potential complications.

The study protocol was approved by the Institutional Review Board of the Second Affiliated Hospital of Xingtai Medical College (Approval no.: KY2024017), and the requirement for informed consent was waived due to the retrospective nature of the study.

*Inclusion criteria*: 1. Patients clinically and laboratory-confirmed as ESRD requiring vascular access surgery; 2. Availability of complete perioperative and follow-up data, including hemodynamic parameters and clinical assessment records; 3. Age ≥ 18 years.
*Exclusion criteria*: 1. Patients with concurrent severe cardiovascular or cerebrovascular events (e.g., recent myocardial infarction or stroke), active systemic infections, or malignancies; 2. Lack of key clinical or imaging data; 3. History of limb amputation or severe peripheral arterial occlusive disease affecting the access site.

The detailed participant screening and exclusion process is illustrated in the Study Flowchart (Supplementary Figure 1).

### 2.3 Diagnostic criteria for DASS

The diagnosis and grading of DASS were established according to the KDOQI Clinical Practice Guideline for Vascular Access: 2019 Update (15), which defines hemodialysis access-induced distal ischemia based on clinical symptoms and hemodynamic severity. This was further supplemented by the diagnostic criteria for hemodynamic assessment outlined in the European Society for Vascular Surgery 2018 Clinical Practice Guidelines (16). In alignment with the latest clinical standards, ultrasound-derived parameters and interventional assessment were employed to confirm the steal phenomenon and evaluate distal perfusion, consistent with contemporary expert consensus on ultrasound-guided vascular access management (17).

Given the retrospective nature of this study, the diagnosis was determined by reviewing patients’ electronic medical records, physical examination findings, and hemodynamic assessment results. The specific diagnostic criteria included:

- *Clinical ischemic symptoms*: Presence of pallor, cold intolerance, numbness, rest pain, or motor dysfunction (e.g., decreased grip strength) in the access-side limb.
- *Hemodynamic evidence*: Documentation of reduced blood flow or retrograde flow in the distal artery via Doppler Ultrasound (DUS) or Digital Subtraction Angiography (DSA).
- *Physical maneuvers*: Documented improvement in distal limb perfusion (e.g., recovery of finger oxygen saturation or enhancement of radial/ulnar artery pulsation) during temporary occlusion of the vascular access (digital compression test).

To ensure diagnostic accuracy and consistency, all cases were independently reviewed by two senior vascular surgeons. Any discrepancies were resolved through discussion or by a third senior expert to reach a final consensus.

### 2.4 Data collection and quality control

Given the retrospective nature of this study, clinical data were retrieved from the patients’ electronic medical records, physical examination findings, and hemodynamic assessment results. To ensure the predictive validity of the baseline characteristics, all clinical data, laboratory parameters, and functional assessments—including the FFS and sarcopenia-related metrics—were collected on the day prior to surgery. Similarly, preoperative hemodynamic indicators were recorded during a standardized evaluation performed within the 24-h window preceding the surgical procedure.

To ensure data accuracy and consistency, all data extraction and entry were performed independently by two researchers in a double-blind manner. Upon completion, a cross-check was conducted. Any ambiguities or inconsistencies were resolved by tracing the original medical records and consulting a third senior researcher to reach a consensus.

#### 2.4.1 Demographic and clinical baseline characteristics

Demographic data, including age, sex, and BMI, were recorded. Primary diseases (e.g., diabetic nephropathy, hypertensive nephrosclerosis), dialysis vintage, and relevant medical history were also documented.

#### 2.4.2 Laboratory indicators

Peripheral blood biochemical results within 1 week prior to surgery were collected, including hemoglobin (Hb), serum albumin (ALB), and urea clearance rate (single-pool Kt/V). Additionally, baseline blood pressure parameters, including diastolic blood pressure (DBP) and distal artery pressure (DAP), were recorded.

#### 2.4.3 Functional assessment

Sarcopenia was defined and diagnosed according to the Asian Working Group for Sarcopenia 2019 (AWGS 2019) criteria, incorporating the evaluation of muscle mass, muscle strength, and physical function (18, 19).

##### 2.4.3.1 Muscle mass

Appendicular skeletal muscle mass was measured using bioelectrical impedance analysis. The appendicular skeletal muscle index was calculated as the sum of lean mass in the four limbs divided by the height squared (kg/m^2^). In accordance with AWGS 2019 cutoffs, low muscle mass was defined as an ASMI < 7.0 kg/m^2^ for men and < 5.7 kg/m^2^ for women.

##### 2.4.3.2 Muscle strength

Handgrip strength was assessed using a standardized handheld dynamometer. Participants performed at least two maximal isometric contractions with their dominant hand, and the maximum value was recorded. Low muscle strength was defined as < 28 kg for men and < 18 kg for women.

##### 2.4.3.3 Physical function

Physical performance was evaluated using the 6-meter usual gait speed test. Participants walked 6 meters at their habitual pace, and a gait speed < 1.0 m/s was categorized as low physical performance.

##### 2.4.3.4 Diagnostic criteria

Following the AWGS 2019 diagnostic algorithm, sarcopenia was diagnosed when a participant exhibited low muscle mass in combination with either low muscle strength or low physical performance.

##### 2.4.3.5 Frailty assessment

Evaluated using the Fried Frailty Phenotype (FFS) scale, covering five dimensions: weight loss, exhaustion, grip strength, gait speed, and physical activity. A total score of ≥ 3was defined as frailty (20, 21).

#### 2.4.4 Hemodynamic and imaging parameters

All vascular assessments were performed by two senior sonographers using a high-resolution Doppler ultrasound system.

Observation indicators: Blood flow velocity (BFV), vessel diameter (VD), and ankle-brachial index (ABI).
Quality control protocol: Patients were placed in a supine position with limbs naturally extended. Measurements were taken at the distal artery of the vascular access. Each indicator was measured three times consecutively, and the arithmetic mean was used to minimize measurement bias.

#### 2.4.5 Surgical factors

Information regarding the type of vascular access (e.g., AVF, AVG), surgical anastomosis technique, site of arterial harvest, and the surgeon’s years of experience (used as a covariate to control for technical bias) was recorded.

### 2.5 Management of missing data

The proportion of missing data for all candidate predictors ranged from 0 to 6.8% (detailed in Supplementary Table 1). Variables with a missing rate exceeding 0% were assumed to be missing at Random. To minimize bias and maintain statistical power, we employed Multiple Imputation by Chained Equations to create five imputed datasets. The final analysis was performed by pooling the results from these datasets according to Rubin’s rules (22). No participants were excluded solely due to missing clinical variables, ensuring a final analytic cohort of 324 patients.

### 2.6 Statistical analysis

All statistical analyses were performed using R software (version 4.5.2). All tests were two-tailed, and a *p*-value < 0.05 was considered statistically significant.

#### 2.6.1 Baseline characteristics and descriptive statistics

Continuous variables were expressed as mean ± standard deviation or median (interquartile range, IQR) based on their distribution. Intergroup comparisons were performed using the Student’s t-test or the Mann–Whitney U test, as appropriate. Categorical variables were presented as frequencies and percentages (%), with comparisons conducted using the Chi-square test or Fisher’s exact test.

#### 2.6.2 Feature selection and model interpretability

To mitigate potential multicollinearity and identify the most relevant predictors for DASS, variables with statistical significance in the intergroup comparisons were initially selected as candidate predictors. Subsequently, the LASSO logistic regression was employed for variable selection. Prior to modeling, all continuous variables were standardized using Z-score transformation. The optimal penalty parameter (*λ*) was determined via 10-fold cross-validation. We reported both λ_min_ (minimum cross-validation error) and λ_1se_ (the simplest model within one standard error of the minimum) to balance model fit and parsimony.

To enhance model interpretability, SHAP analysis was utilized to assess the contribution of each feature to the predictive output. SHAP summary plots and partial dependence plots were generated to visualize the direction and relative importance of key variables on DASS risk, ensuring clinical transparency.

#### 2.6.3 Logistic regression and robustness assessment

Univariate and multivariable logistic regression analyses were conducted to identify independent risk factors for DASS. Variables selected by LASSO regression were first entered into the univariate analysis, and those with *p* < 0.05 were subsequently included in the multivariable model. Results were reported as odds ratios (OR) with 95% confidence intervals (CI).

To evaluate model stability and the impact of potential confounding, sensitivity analyses were performed by excluding participants with extreme measurements. Additionally, E-values were calculated to quantify the potential influence of unmeasured confounders. Multicollinearity among predictors was assessed using the Variance Inflation Factor (VIF), and model performance was evaluated using the Nagelkerke *R*^2^ and Brier score.

#### 2.6.4 BMI stratification and interaction analysis

Participants were categorized into three groups based on BMI levels: underweight (<18.5 kg/m^2^), normal weight (18.5–24.9 kg/m^2^), and overweight/obese (≥ 25 kg/m^2^). Interaction models were constructed by including product terms (e.g., Predictor × BMI_category_) in the multivariable logistic regression. To enhance the stability of the model and minimize multicollinearity, continuous predictors involved in interaction terms were mean-centered before the creation of product terms.

Interaction models were constructed to evaluate the effect modification of BMI. Interaction effects were reported as OR_interaction_. Furthermore, additive interactions were assessed by calculating the Relative Excess Risk due to Interaction (RERI), the Attributable Proportion (AP), and the Synergy Index (S).

#### 2.6.5 Assessment of non-linear relationships

RCS were employed to evaluate potential non-linear associations between continuous independent risk factors (BMI, DAP, CKD duration, and FFS) and DASS risk. Four knots were positioned at the 5th, 35th, 65th, and 95th percentiles of the variable distributions. Using the lowest risk level or the median as the reference value, the trends between variables and DASS risk were visualized. Wald χ^2^ tests were used to assess overall associations and non-linearity. A non-linearity *P*_value_ < 0.05 indicated a significant non-linear relationship.

#### 2.6.6 Model construction and performance evaluation

A final predictive model was developed, and ROC curves were generated. The predictive efficacy was quantified by the Area under the Curve (AUC). Model discrimination was assessed using the C-index and AUC, while calibration was evaluated via calibration curves, including the calibration slope, intercept, and Brier score. Finally, internal validation was performed using 1,000-iteration bootstrap resampling and 10-fold cross-validation to assess the robustness and generalizability of the model.

## 3 Results

### 3.1 Baseline clinical, laboratory, and procedural characteristics

A total of 324 patients were included, with 86 (26.5%) diagnosed with DASS and 238 (73.5%) in the non-DASS group (Table 1).

**Table 1:** Comparison of demographic and clinical characteristics between the DASS and non-DASS groups in patients undergoing hemodialysis vascular access surgery.

Variables	Overall (*n* = 324)	Non-DASS Goup (*n* = 238)	DASS Goup (*n* = 86)	*P*_value_
Clinical characteristics				
Age (years)	55.52 ± 15.50	54.10 ± 15.68	59.44 ± 1 4.34	<0.001
Sex (Male, N%)	210 (64.8%)	150 (63.0%)	60 (69.8%)	0.533
BMI (kg/m^2^)	24.74 ± 4.38	25.54 ± 4.42	22.54 ± 3.45	<0.001
CKD Duration (years)	10.60 ± 3.23	12.21 ± 2.61	10.02 ± 3.24	<0.001
Diabetic Nephropathy (Yes, N %)	120 (37.0%)	72 (30.3%)	48 (55.8%)	<0.001
Hypertensive Nephropathy (Yes, N %)	98 (30.2%)	78 (32.8%)	20 (23.3%)	0.258
Other Kidney Diseases (Yes, N %)	65 (20.1%)	37 (15.5%)	28 (32.6%)	0.003
Dialysis Rate (mL/kg/h)	264.52 ± 48.91	263.36 ± 50.74	267.74 ± 43.56	0.776
spKt/V	1.45 ± 0.29	1.48 ± 0.29	1.37 ± 0.26	0.016
Systolic BP (mmHg)	137.29 ± 20.90	135.83 ± 21.43	141.31 ± 18.90	0.113
Diastolic BP (mmHg)	86.41 ± 14.14	84.68 ± 13.99	91.20 ± 13.50	0.001
Hemoglobi (g/dL)	11.22 ± 1.89	11.43 ± 1.84	10.64 ± 1.92	0.004
Serum Albumin (g/L)	36.72 ± 4.68	37.18 ± 4.72	35.43 ± 4.36	0.012
Fried Criteria (Items)	2.60 ± 1.21	2.08 ± 0.82	4.06 ± 0.87	<0.001
Sarcopenia (Yes, N %)	106 (32.7%)	88 (37.0%)	68 (79.1%)	0.025
Imaging and physiological parameters				
Blood Flow Velocity (mL/min)	971.21 ± 328.19	1037.92 ± 335.87	786.58 ± 218.66	<0.001
Vessel diameter (mm)	6.66 ± 1.80	7.01 ± 1.77	5.70 ± 1.51	<0.001
ABI	0.97 ± 0.19	1.00 ± 0.18	0.88 ± 0.18	<0.001
DAP (mmHg)	86.38 ± 18.82	90.85 ± 18.17	74 ± 14.66	<0.001
Surgical techniques				
AVF end-to-side anastomosis (Yes, N %)	220 (67.9%)	170 (71.4%)	50 (58.1%)	0.078
AVF side-to-side anastomosis (Yes, N %)	50 (15.4%)	30 (12.6%)	20 (23.3%)	0.064
AVG (Yes, N %)	54 (16.7%)	38 (16.0%)	16 (18.6%)	0.854
Vessel selection				
Brachial Artery (Yes, N %)	180 (55.6%)	130 (54.6%)	50 (58.1%)	0.854
Radial Artery (Yes, N %)	144 (44.4%)	108 (45.4%)	36 (41.9%)	0.854
Operator EXP (years)	11.29 ± 5.02	12.25 ± 5.12	8.63 ± 3.61	<0.001

Data are presented as mean ± standard deviation (SD) for continuous variables and as number (percentage) for categorical variables. *P*_value_ indicate the comparison between the DASS and non-DASS groups, with statistical significance defined as *p* < 0.05 (shown in bold). DASS, Depression, Anxiety and Stress Scale; BMI, body mass index; CKD, chronic kidney disease; spKt/V, single-pool Kt/V; BP, blood pressure; ABI, ankle-brachial index; DAP, dialysate arterial pressure; AVF, arteriovenous fistula; AVG, arteriovenous graft.

Compared to the non-DASS group, patients with DASS were significantly older (59.44 ± 14.34 vs. 54.10 ± 15.68 years, *p* < 0.001), had a lower BMI (22.54 ± 3.45 vs. 25.54 ± 4.42 kg/m^2^, *p* < 0.001), and a longer CKD duration (14.06 ± 6.94 vs. 10.36 ± 6.14 years, *p* < 0.001). A higher prevalence of diabetic nephropathy and other renal primary diseases was also observed in the DASS group (*p* < 0.01).

Regarding laboratory and clinical assessments, the DASS group showed significantly lower levels of spKt/V, hemoglobin, and serum albumin, alongside higher diastolic blood pressure and FFS. The incidence of sarcopenia was notably higher in DASS patients (*p* < 0.05).

Hemodynamic and imaging evaluations revealed that the DASS group had lower blood flow velocity, smaller vessel diameter, reduced ABI, and lower DAP (all *p* < 0.001). While vascular access type and arterial selection were comparable between groups (*p* > 0.05), the surgical experience of the operators was significantly lower in the DASS group (8.63 ± 3.61 vs. 12.25 ± 5.12 years, *p* < 0.001).

### 3.2 Feature selection and risk factor identification via LASSO regression

**Figure 2:**
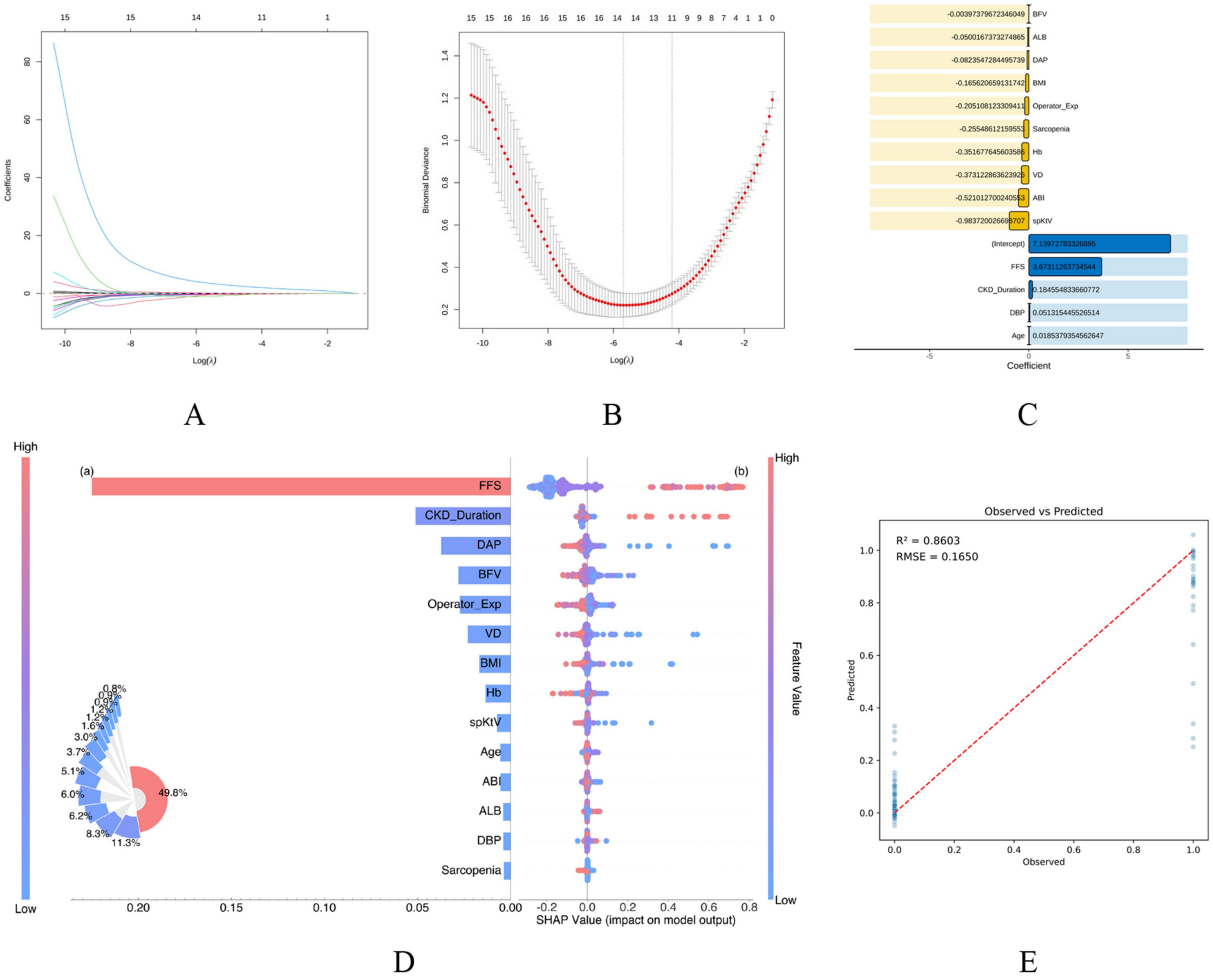
LASSO regression and SHAP analysis for the identification and interpretation of independent predictors for DASS in ESRD patients. **(A)** LASSO coefficient profiles: variation in the coefficients of 15 baseline clinical variables relative to the log (*Λ*). **(B)** Tuning parameter (λ) selection: optimal λ was determined using 10-fold cross-validation. The vertical dashed line indicates the value of λ that yields the minimum mean cross-validated error. **(C)** Variable importance ranking: selection of features with non-zero coefficients at the optimal λ, identified as independent predictors of DASS. **(D)** SHAP summary plot: evaluation of the global contribution of each feature variable. The plot illustrates the distribution of SHAP values and the directional impact of feature values on DASS risk. Notably, Fried Frailty Score emerged as a predominant feature with a strong positive association with the outcome. **(E)** SHAP observed-versus-predicted plot: assessment of model calibration and stability. The high concordance between predicted probabilities and actual DASS occurrence, supported by an *R*^2^ of 0.8603, indicates robust predictive performance and interpretability.

To identify independent predictors of dialysis DASS in patients with ESRD, we constructed a LASSO logistic regression model. This model incorporated clinically significant baseline variables that exhibited marked differences between groups (refer to Supplementary Table S2). By optimizing the tuning parameter through cross-validation, 14 variables with non-zero coefficients were retained at *λ* ≈ −8 (Figures 2A–C). Notably, the Fried frailty criteria emerged as the most potent predictor, demonstrating a robust positive correlation with DASS risk (coefficient = 3.67; Figure 2C).

To enhance model interpretability, we performed SHAP analysis, which further substantiated the clinical dominance of the Fried criteria. The SHAP summary plot indicated that the Fried criteria accounted for 49.8% of the total feature importance, with elevated frailty scores significantly escalating the probability of DASS (Figure 2D). Furthermore, partial dependence plots elucidated complex interaction effects between frailty scores and other parameters—such as BFV, BMI, and CKD duration—underscoring the central role of systemic frailty in modulating DASS risk (Supplementary Figures S2A–N). Finally, the SHAP observed-versus-predicted analysis revealed high concordance between the predicted risk and clinical outcomes (*R*^2^ = 0.8603; Figure 2E), confirming the model’s superior stability and predictive accuracy.

This integrated machine-learning approach successfully identified a refined subset of predictors with high statistical and clinical relevance, providing a rigorous framework for risk stratification in ESRD patients.

### 3.3 Multivariable logistic regression and model performance

Building upon the LASSO-based feature selection, we constructed a multivariable logistic regression model to identify independent determinants of DASS in patients with ESRD. The analysis identified several statistically significant predictors, including BMI (OR = 0.70; 95% CI: 0.54–0.92; *p* = 0.011), CKD duration (OR = 1.25; 95% CI: 1.06–1.47; *p* = 0.008), sarcopenia (OR = 1.42; 95% CI: 1.05–1.92; *p* = 0.024), DAP (OR = 0.88; 95% CI: 0.80–0.96; *p* = 0.004), Operator experience (OR = 0.70; 95% CI: 0.55–0.89; *p* = 0.004), and FFS (OR = 2.35; 95% CI: 1.10–3.60; *p* = 0.018; Table 2).

**Table 2:** Univariate and multivariate logistic regression analysis of risk factors associated with DASS in ESRD patients.

Independent variable	Univariate OR (95% CI)	*P*_value_	Multivariate OR (95% CI)	*P*_value_
BMI	0.84 (0.79, 0.90)	<0.001	0.70 (0.54, 0.92)	0.011
CKD duration	1.09 (1.05, 1.14)	<0.001	1.25 (1.06, 1.47)	0.008
BFV	1.00 (1.00, 1.00)	<0.001	0.99 (0.99, 1.02)	0.187
Hb	0.80 (0.70, 0.91)	0.001	0.67 (0.38, 1.18)	0.166
Sarcopenia	1.78 (1.22, 2.59)	0.003	1.42 (1.05, 1.92)	0.024
DAP	0.94 (0.93, 0.96)	<0.001	0.88 (0.80, 0.96)	0.004
DBP	1.03 (1.02, 1.05)	<0.001	1.08 (1.00, 1.15)	0.43
VD	0.63 (0.54, 0.74)	<0.001	0.73 (0.37, 1.41)	0.344
ALB	0.92 (0.87, 0.97)	0.003	0.89 (0.70, 1.13)	0.339
Operator experience	0.85 (0.80, 0.90)	<0.001	0.70 (0.55, 0.89)	0.004
Age	1.02 (1.01, 1.04)	0.007	1.05 (0.97, 1.12)	0.212
ABI	0.26 (0.16, 0.41)	<0.001	0.85 (0.70, 1.03)	0.428
spKt/V	0.28 (0.11, 0.67)	0.005	0.10 (0.01, 1.05)	0.055
FFS	5.21 (3.80, 6.62)	0.04	2.35 (1.10, 3.60)	0.018

The multivariate logistic regression model was constructed using variables that showed statistical significance (*p* < 0.05) in the univariate analysis. Sarcopenia was treated as a categorical variable, with “No” assigned as the reference group (value = 1). FFS refers to the Fried Frailty Score. OR, odds ratio; CI, confidence interval; BMI, body mass index; CKD, chronic kidney disease; BFV, blood flow velocity; Hb, hemoglobin; DAP, dialysate arterial pressure; DBP, diastolic blood pressure; VD, vessel diameter; ALB, albumin; ABI, ankle-brachial index; spKt/V, single-pool Kt/V.

The overall performance of the predictive model was evaluated using the Nagelkerke R^2^ and the Brier score. A Nagelkerke *R*^2^ of 0.38 indicated that the selected variables accounted for approximately 38% of the variance in DASS occurrence. Furthermore, a Brier score of 0.14 demonstrated high overall predictive accuracy and favorable calibration, suggesting a close alignment between predicted probabilities and observed clinical outcomes.

To ensure the reliability of the estimated coefficients, multicollinearity was assessed using the VIF. All retained predictors exhibited VIF values < 2, indicating the absence of significant collinearity and confirming the statistical robustness of the model. Collectively, these findings validate the identified variables as independent, clinically relevant risk factors, providing a reliable quantitative framework for DASS risk stratification in the ESRD population.

### 3.4 Sensitivity analysis of multivariable logistic regression results

To evaluate the robustness of our primary findings, a sensitivity analysis was performed by excluding extreme values to re-examine the independent risk factors for DASS in patients with ESRD. The results demonstrated that the directionality and statistical significance of the associations remained highly consistent with the original multivariable regression model (Table 3).

**Table 3:** Sensitivity analysis and E-value assessment of independent risk factors for DASS in ESRD patients.

Variable	OR after excluding extremes (95% CI)	*P*_value_	E_value_
CKD duration	1.23 (1.05, 1.46)	0.01	1.95
Sarcopenia	1.40 (1.04, 1.91)	0.03	2.17
DAP	0.87 (0.79, 0.95)	<0.01	1.64
FFS	2.33 (1.09, 3.57)	0.02	2.77
BMI	0.69 (0.53, 0.91)	0.01	2.00
Operator experience	0.55 (0.45, 0.67)	<0.01	1.32

Sensitivity analysis was conducted by excluding extreme values (*n* = 6, representing the top and bottom 1% of the distribution for continuous variables such as BMI and DAP) to verify the stability of the multivariate logistic regression model. E-value represents the minimum strength of association that an unmeasured confounder would need to have with both the exposure and the outcome to nullify the observed association. Higher E-values indicate that the observed associations are more robust against potential unmeasured confounding. OR, odds ratio; CI, confidence interval; CKD, chronic kidney disease; DAP, dialysate arterial pressure; FFS, Fried Frailty Score; BMI, body mass index.

Specifically, CKD duration persisted as a significant risk factor after the exclusion of outliers (OR = 1.23; 95% CI: 1.05–1.46; *p* = 0.01; E-value = 1.95). The calculated E-value indicates that an unmeasured confounder would require a relatively strong association with both the exposure and the outcome to nullify the observed effect. Similar stability was observed for sarcopenia (OR = 1.40; 95% CI: 1.04–1.91; *p* = 0.03; E-value = 2.17), DAP (OR = 0.87; 95% CI: 0.79–0.95; *p* < 0.01; E-value = 1.64), FFS (OR = 2.33; 95% CI: 1.09–3.57; *p* = 0.02; E-value = 2.77), BMI (OR = 0.69; 95% CI: 0.53–0.91; *p* = 0.01; E-value = 2.00), and operator experience (OR = 0.55; 95% CI: 0.45–0.67; *p* < 0.01; E-value = 1.32), all of which retained their original effect sizes and statistical significance.

Furthermore, to assess the impact of missing data, we compared the results derived from the MICE-imputed dataset (*N* = 324) with those from the complete case analysis (*n* = 286). As shown in Supplementary Table 3, the effect sizes and statistical significance across both approaches were highly concordant, further validating the reliability of our findings.

In summary, these sensitivity analyses confirm that the multivariable logistic regression results are robust and minimally influenced by extreme observations or data imputation strategies. The E-values suggest that the identified associations are unlikely to be entirely attributable to unmeasured confounding, reinforcing the reliability of our risk factor identification.

### 3.5 Stratified analysis by BMI categories

To investigate whether BMI modulates the risk profile of DASS, we performed a stratified analysis across three categories (Low: <18.5; Normal: 18.5–24.9; High: ≥ 25). This analysis followed the sensitivity tests to ensure that the findings reflect stable biological associations rather than the influence of extreme values.

In the low-BMI subgroup (Table 4), CKD duration (OR = 1.35; 95% CI: 1.15–1.59; *p* < 0.001), sarcopenia (OR = 1.48; 95% CI: 1.18–1.86; *p* = 0.001), and FFS (OR = 1.25; 95% CI: 1.08–1.44; *p* = 0.003) were significantly associated with an increased DASS risk, while higher DAP exerted a protective effect (OR = 0.80; 95% CI: 0.68–0.94; *p* = 0.007).

**Table 4:** Stratified analysis of independent risk factors for DASS across different BMI categories.

BMI group	Variable	OR	95% CI	*P*_value_
Low BMI (<18.5 kg/m^2^)	CKD duration	1.35	1.15, 1.59	<0.001
	Sarcopenia	1.48	1.18, 1.86	0.001
	DAP	0.8	0.68, 0.94	0.007
	Operator experience	1.01	0.87, 1.18	0.9
	FFS	1.25	1.08, 1.44	0.003
Normal BMI (18.5–24.9 kg/m^2^)	CKD duration	1.22	1.08, 1.38	0.002
	Sarcopenia	1.32	1.08, 1.61	0.007
	DAP	0.85	0.74, 0.97	0.018
	Operator experience	1.03	0.90, 1.18	0.65
	FFS	1.18	1.04, 1.33	0.01
High BMI (≥25 kg/m^2^)	CKD duration	1.08	0.95, 1.22	0.22
	Sarcopenia	1.15	0.92, 1.44	0.21
	DAP	0.9	0.78, 1.05	0.17
	Operator experience	1.02	0.88, 1.18	0.78
	FFS	1.1	0.95, 1.28	0.19

Stratified analysis was conducted based on BMI categories to assess the stability of the predictive variables. Statistical significance (*p* < 0.05) is highlighted in bold. OR, odds ratio; CI, confidence interval; BMI, body mass index; CKD, chronic kidney disease; DAP, dialysate arterial pressure; FFS, Fried Frailty Score.

A consistent pattern was observed in the normal-BMI subgroup (Table 4), where CKD duration, sarcopenia, FFS, and DAP remained significant predictors (all *p* < 0.05). Notably, Operator_Exp did not significantly influence outcomes in either the low or normal BMI cohorts (*p* > 0.05).

In contrast, the predictive value of these variables was markedly attenuated in the high-BMI subgroup (Table 4), where none of the factors—including CKD duration, sarcopenia, DAP, and FFS—retained statistical significance (all *p* > 0.05).

These results reveal a stratified divergence in risk factors, suggesting that the impact of systemic frailty and vascular parameters on DASS is more pronounced in patients with low-to-normal BMI. This interaction highlights BMI as a potential effect modifier, reinforcing the necessity of BMI-specific risk stratification for individualized DASS prediction.

### 3.6 Interaction analysis of BMI and clinical risk factors

To further elucidate the interplay between BMI and other independent risk factors, both multiplicative and additive interaction analyses were performed (Table 5). The results revealed significant multiplicative interactions between BMI and several key variables, including sarcopenia (OR_interactio_ = 1.30; *p* = 0.012), DAP (OR_interaction_ = 0.88; *p* = 0.025), CKD duration (OR_interaction_ = 1.12; *p* = 0.015), and FFS (OR_interaction_ = 1.15; *p* = 0.018).

**Table 5:** Measures of additive and multiplicative interaction between BMI and other risk factors for DASS.

Interaction	ORinteraction (95% CI)	*P*_value_	RERI	AP	S
BMI × Sarcopenia	1.30 (1.05, 1.60)	0.012	0.17	0.13	2.42
BMI × DAP	0.88 (0.78, 0.98)	0.025	0.18	0.33	−3.40
BMI × CKD Duration	1.12 (1.02, 1.23)	0.015	0.22	0.43	−2.10
BMI × FFS(Frailty)	1.15 (1.03, 1.29)	0.018	0.19	0.08	1.15

OR (95% CI) represents the multiplicative interaction (product term) in the logistic regression model. RERI: RERI > 0 indicates a significant additive synergistic effect. AP: Represents the proportion of the combined effect that is due to the interaction. S: S > 1 indicates a positive additive interaction. DAP: Distal Arterial Pressure; FFS: Fried Frailty Score.

In addition to multiplicative effects, significant additive interactions were observed across all tested pairs. The RERI was consistently greater than zero for BMI interacting with sarcopenia (RERI = 0.17), DAP (RERI = 0.18), CKD duration (RERI = 0.22), and FFS (RERI = 0.19), suggesting that the combined presence of these factors and low BMI given the previously established protective role of BMI results in a risk that exceeds the sum of their individual effects. Specifically, the S for the BMI × sarcopenia (S = 2.42) and BMI × FFS (S = 1.15) interactions further confirmed a positive synergistic effect on DASS risk. The AP indicated that the interaction between BMI and CKD duration accounted for the highest proportion of the combined effect (AP = 43%).

These findings suggest that BMI acts as a significant effect modifier, whereby the detrimental impacts of frailty, sarcopenia, and prolonged CKD duration on DASS risk are compounded in specific BMI strata. Such synergistic relationships emphasize the complexity of DASS pathogenesis and the necessity of integrated risk assessment.

### 3.7 Restricted cubic spline analysis and threshold identification

**Figure 3:**
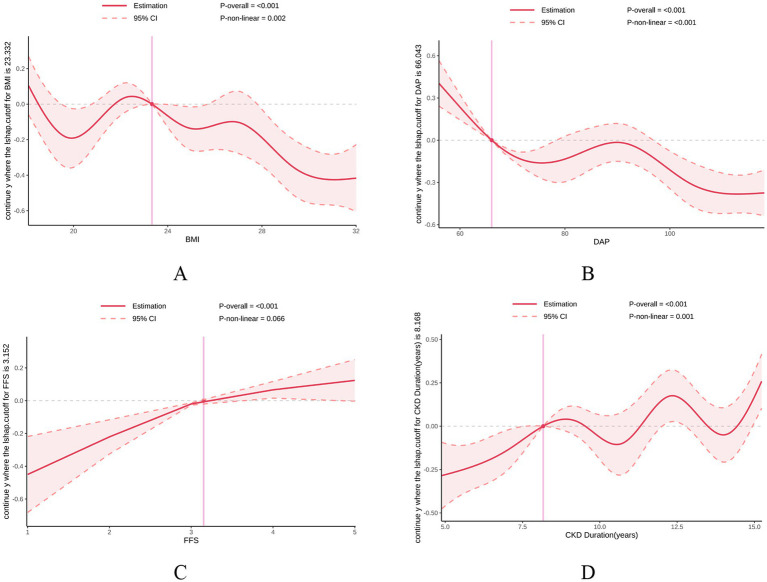
RCS analysis of continuous risk factors for DASS in ESRD patients. The solid red lines represent the estimated odds ratios (or transformed risk scores), and the pink shaded areas indicate the 95% CIs. The vertical pink dashed lines denote the reference cutoff points where the SHAP-based risk trajectory transitions. *P*-overall represents the global significance of the variable in the model, and *P*-non-linear indicates the statistical significance of the non-linear dose–response relationship. **(A)** BMI: A significant non-linear association was observed (*P*-non-linear = 0.002), with DASS risk fluctuating across BMI levels and a reference point at 23.332 kg/m^2^. **(B)** DAP: A strong non-linear trend (*P*-non-linear < 0.001) suggests that DASS risk decreases as DAP increases up to 66.043 mmHg, after which the risk trend stabilizes. **(C)** FFS: The association was predominantly linear (*P*-non-linear = 0.066), with DASS risk increasing steadily as Fried frailty scores rise (reference point: 3.152). **(D)** CKD duration: A complex non-linear relationship was identified (*P*-non-linear = 0.001), showing multiple risk fluctuations as the duration of CKD increases (reference point: 8.168 years). BMI, body mass index; CKD, chronic kidney disease; DAP, dialysate arterial pressure; FFS, fried frailty status.

To characterize the potential non-linear dose–response relationships between continuous predictors and DASS risk, RCS regression was performed with knots at the 10th, 50th, and 90th percentiles (Figure 3). All candidates exhibited significant overall associations with DASS (P_overall_ < 0.001).

Significant non-linearity was observed for BMI, DAP, and CKD duration (all P_non-linear_ < 0.05), while FFS exhibited a robust linear risk escalation (P_non-linear_ = 0.066). Key clinical thresholds and trajectories were identified as follows:

BMI (Figure 3A): A threshold was identified at 23.3 kg/m^2^, below which DASS risk increased sharply as BMI declined, whereas the risk remained low and stable in the higher BMI range.

DAP (Figure 3B): A prominent threshold effect occurred at 66 mmHg; risk escalated precipitously below this level but reached a protective plateau once DAP exceeded 70–80 mmHg.

CKD Duration (Figure 3D): A turning point emerged at 8.2 years, after which the risk trajectory showed a fluctuating but significant upward trend, indicating heightened vulnerability in long-term ESRD patients.

FFS (Figure 3C): Consistent with its linear nature, FFS showed a steady risk increase; any score elevation beyond the reference (FFS = 3) was associated with a proportional rise in DASS probability.

These findings highlight specific clinical benchmarks—BMI < 23.3 kg/m^2^, DAP < 66 mmHg, and CKD duration > 8.2 years—as critical indicators for intensified risk stratification in patients undergoing vascular access surgery.

### 3.8 Performance and validation of the predictive model

**Figure 4:**
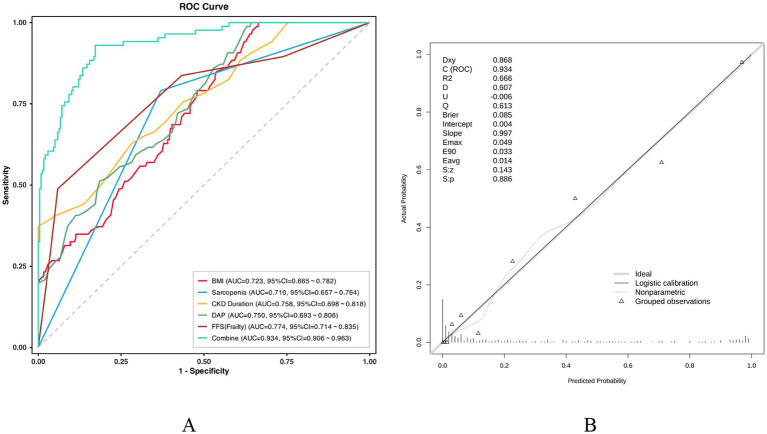
ROC curves and calibration plot analysis of the predictive model and its individual components. **(A)** Comparative analysis of ROC curves. This plot illustrates the predictive performance of BMI, Sarcopenia, CKD Duration, DAP, FFS (Frailty), and the integrated model (Combine) for the target outcome. 1. The AUC and its corresponding 95% CI for each variable are presented in the legend. 2. The Combine model (cyan solid line) demonstrates the highest predictive accuracy with an AUC of 0.934 (95% CI: 0.906–0.963). **(B)** Calibration curve of the integrated model. The x-axis represents the predicted probability, while the y-axis represents the actual observed probability. 1. The Ideal line (gray dashed line) represents a perfect match between predicted and actual probabilities. 2. Both the Logistic calibration (solid line) and Nonparametric (dotted line) curves closely align with the ideal line, indicating excellent calibration. 3. Statistical indices show a C-index (AUC) of 0.934, a calibration slope of 0.997, an intercept of 0.004, and a Brier score of 0.085, further confirming high consistency between the model’s predictions and actual observations. BMI, body mass index; CKD, chronic kidney disease; DAP, dialysate arterial pressure; FFS, fried frailty status; AUC, area under the curve; ROC, receiver operating characteristic curve.

The multivariable logistic regression model, incorporating BMI, sarcopenia, CKD duration, DAP, and FFS, demonstrated superior discriminative performance. The model yielded an impressive AUC of 0.934 (95% CI: 0.906–0.963), which was significantly higher than that of any individual predictor (AUC range: 0.710–0.774; Figure 4A). To ensure the robustness of these findings and adjust for potential overfitting, internal validation was performed using 1,000-iteration bootstrap resampling, resulting in a corrected C-index of 0.931 (95% CI: 0.926–0.934). The generalizability of the model was further rigorously evaluated via 10-fold cross-validation (Supplementary Table 4). Throughout the 10 iterations, the model exhibited high stability, with a mean AUC of 0.934 (range: 0.927–0.945) in the training sets and a mean AUC of 0.926 (range: 0.817–1.000) in the validation sets. Despite minor fluctuations inherent to random data partitioning, the consistent mean performance confirms the model’s strong predictive efficacy across independent data subsets.

Regarding model calibration, the calibration curve illustrated a high degree of concordance between the predicted probabilities and the observed outcomes (Figure 4B). The logistic calibration slope was 0.997 with an intercept of 0.004, closely approximating the ideal parameters of 1.0 and 0, respectively. Furthermore, a low Brier score of 0.085 reinforced the high precision of the model in estimating individual risk. Clinical utility was evidenced through risk stratification, where patients were categorized into high-risk (*n* = 162) and low-risk (*n* = 162) groups based on predicted probabilities. The incidence of the primary outcome was significantly higher in the high-risk group compared to the low-risk group (50.00% vs. 3.09%; *p* < 0.001). These results underscore the model’s capability to effectively differentiate high-risk individuals, providing a reliable foundation for clinical risk management and decision-making.

## 4 Discussion

### 4.1 Summary of main findings

The present study identified a DASS incidence of 26.5% among ESRD patients following vascular access surgery, highlighting the clinical urgency of this complication. By integrating LASSO-SHAP selection and multivariable Logistic regression, we developed and validated a robust prediction model (AUC = 0.934). The core finding is that DASS is not merely a hemodynamic failure but a systemic manifestation involving functional status (FFS, sarcopenia), clinical history (CKD duration), and physical metrics (BMI, DAP). Notably, our study is among the first to quantify the effect modification of BMI on these risk factors, providing a more nuanced framework for preoperative risk assessment.

### 4.2 Functional indicators: the role of frailty and sarcopenia

A pivotal observation in our model is that the FFS and sarcopenia emerged as leading predictors. Traditionally, DASS was viewed through a purely “vessel-centric” lens (23, 24). However, our results suggest that the “host environment” is equally critical. Sarcopenia and high FFS reflect a state of diminished physiological reserve and poor compensatory capacity of the microvasculature (25, 26). In patients with frailty, the peripheral vascular bed may fail to dilate appropriately in response to the “steal” phenomenon, exacerbating distal ischemia. This shifts the focus of preoperative evaluation from simple vascular imaging to a comprehensive geriatric and functional assessment.

### 4.3 Hemodynamic thresholds and surgical experience

Our use of RCS allowed for the identification of specific clinical “tipping points,” such as DAP < 66 mmHg and CKD duration > 8.2 years. The significant association with CKD duration likely reflects the cumulative burden of medial arterial calcification and stiffness, specifically Mönckeberg’s sclerosis (27, 28). This pathological remodeling severely impairs the ability of the collateral circulation to recruit flow and maintain adequate distal perfusion pressure in the face of the “steal” created by the access (29). Furthermore, surgical experience was identified as an independent protective factor. This suggests that the technical nuances of anastomosis—such as the angle of the shunt and precise sizing—play a deterministic role. Experienced surgeons may instinctively modulate the fistula diameter based on the patient’s DAP, thereby balancing the delicate trade-off between access maturation and the preservation of distal limb perfusion.

### 4.4 BMI as an effect modifier: the obesity paradox

A novel contribution of this study is the significant interaction between BMI and other risk factors (*P*_interaction_ < 0.05). We observed that FFS, DAP, and CKD duration were potent predictors primarily in the low/normal BMI group, whereas their impact was attenuated in high-BMI patients. This “obesity paradox” in DASS may be attributed to larger vessel diameters and different soft-tissue pressure environments in obese individuals, which might buffer hemodynamic shifts (30, 31). This suggests that low-BMI patients are a high-vulnerability subgroup where functional and hemodynamic deficits are more likely to translate into clinical ischemia.

### 4.5 Robustness and clinical implications

Robustness and Clinical ImplicationsThe reliability of our results is bolstered by sensitivity analyses and high E-values, suggesting the findings are resilient to unmeasured confounders. From a practical standpoint, our model allows for a “precision medicine” approach. For high-risk patients (50.00% incidence), clinicians should consider pre-operative nutritional support for sarcopenia or choosing distal configurations to minimize steal. For low-BMI patients with high FFS, conservative management of anastomosis size or prophylactic tapering may be warranted to preserve limb integrity.

### 4.6 Strengths and limitations

The strengths include high model interpretability via SHAP values and rigorous validation (C-index = 0.931). However, as a retrospective single-center study, potential selection bias exists. Future multi-center prospective studies are needed to externalize the model’s predictive accuracy across different ethnic populations.

## 5 Conclusion

In conclusion, we have developed a highly discriminative and interpretable model for DASS risk prediction. By identifying DAP < 66 mmHg, CKD > 8.2 years, and low BMI as key risk indicators, and highlighting the profound impact of frailty, our findings advocate for a multidisciplinary approach to vascular access surgery that prioritizes functional and hemodynamic optimization.

## Data Availability

The raw data supporting the conclusions of this article will be made available by the authors, without undue reservation.

## Glossary

Esrd: End-Stage Renal Disease
AVF: Arteriovenous Fistula
AVG: Arteriovenous Graft
DASS: Dialysis Access Steal Syndrome
BMI: Body Mass Index
SHAP: SHapley Additive exPlanations
RCS: Restricted Cubic Splines
LASSO: Least Absolute Shrinkage and Selection Operator
ROC: Receiver Operating Characteristic
DUS: Doppler Ultrasound
DSA: Digital Subtraction Angiography
Hb: Hemoglobin
ALB: Serum Albumin
DBP: Diastolic Blood Pressure
DAP: Distal Artery Pressure
FFS: Fried Frailty Score
BFV: Blood Flow Velocity
VD: Vessel Diameter
ABI: Ankle-Brachial Index
OR: Odds Ratios
CI: Confidence Intervals
VIF: Variance Inflation Factor
RERI: Risk Due to Interaction
AP: Attributable Proportion
S: The Synergy Index
AUC: Area under the Curve
