# Supplementary material for: Development of a risk prediction model for dialysis access steal syndrome: exploring the interaction and modifying role of BMI
Source: Front Public Health. 2026 Apr 7;14:1790958. doi: 10.3389/fpubh.2026.1790958 (PMC13095675; doi:10.3389/fpubh.2026.1790958)
Supplement: Supplementary file 1 [file Supplementary_file_1.docx]

**Supplementary**

| 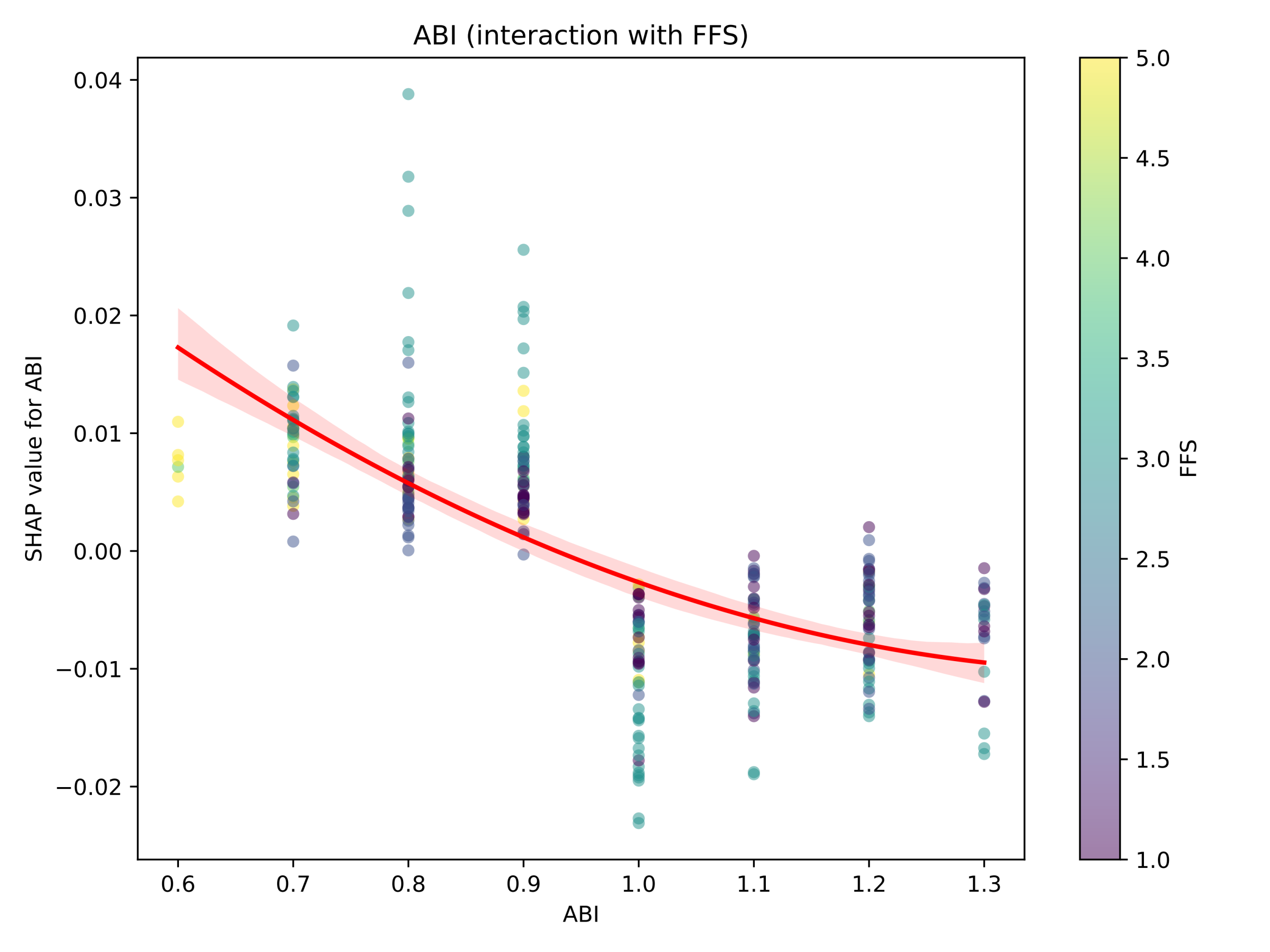 | 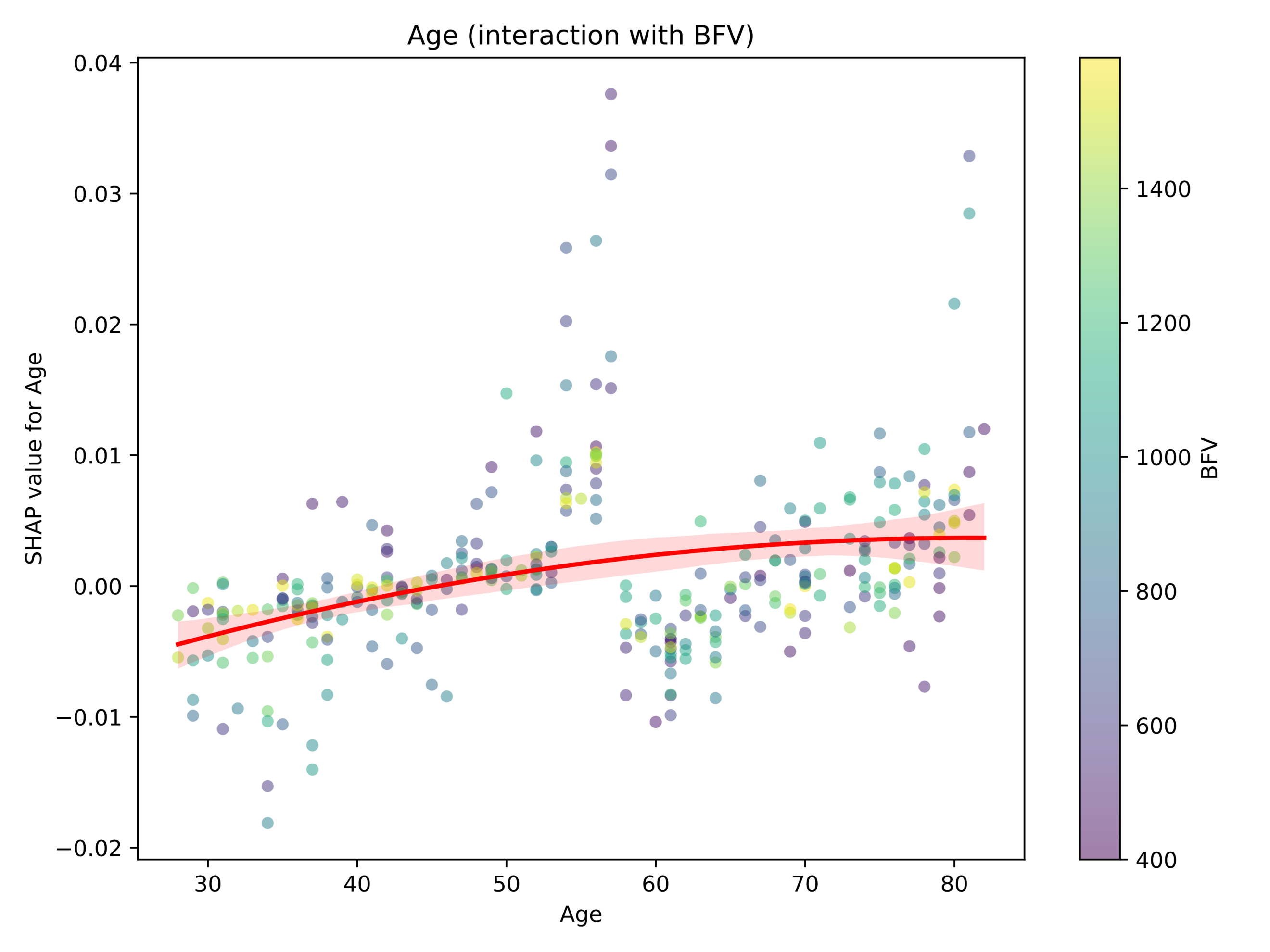 | 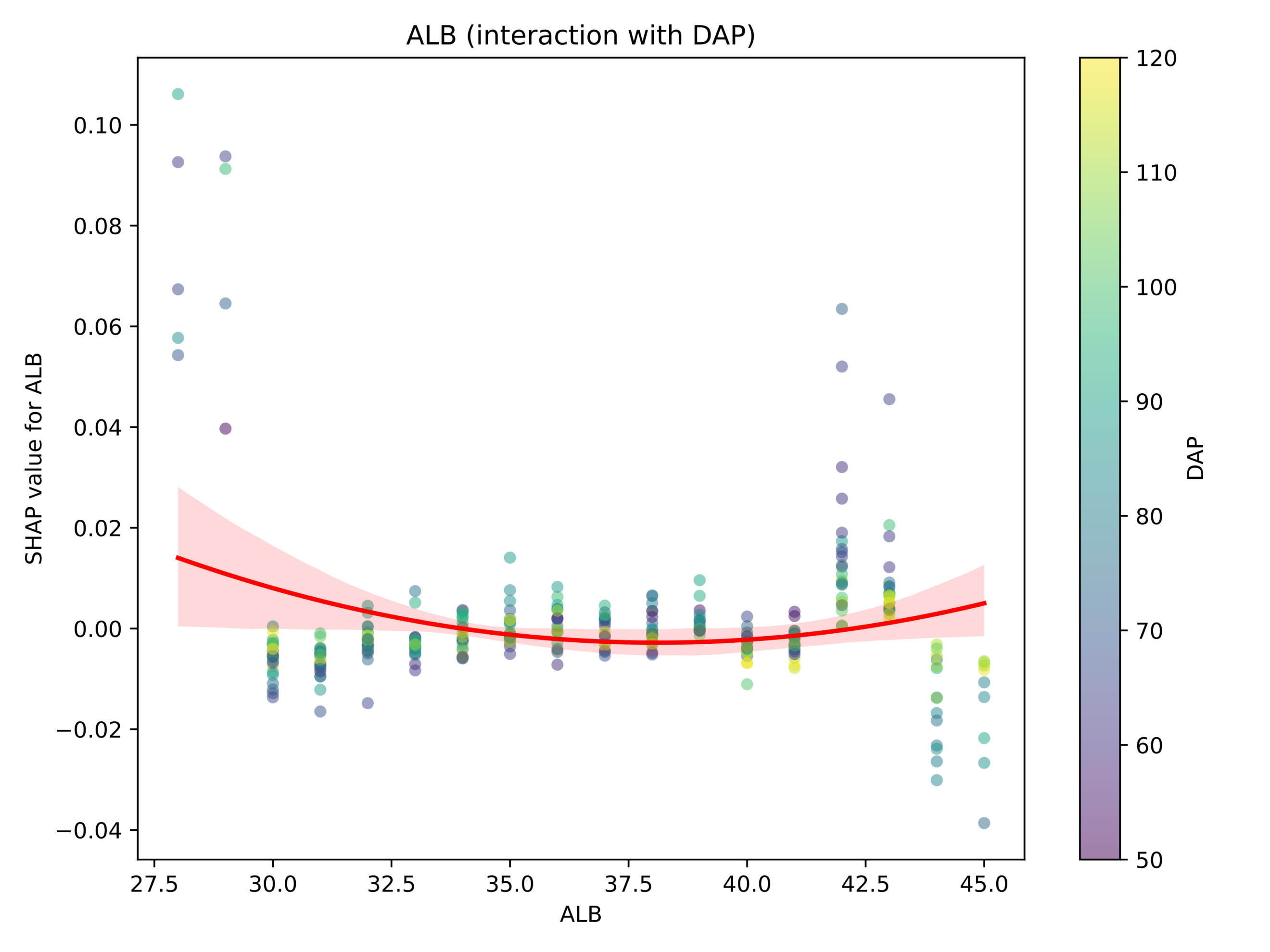 | 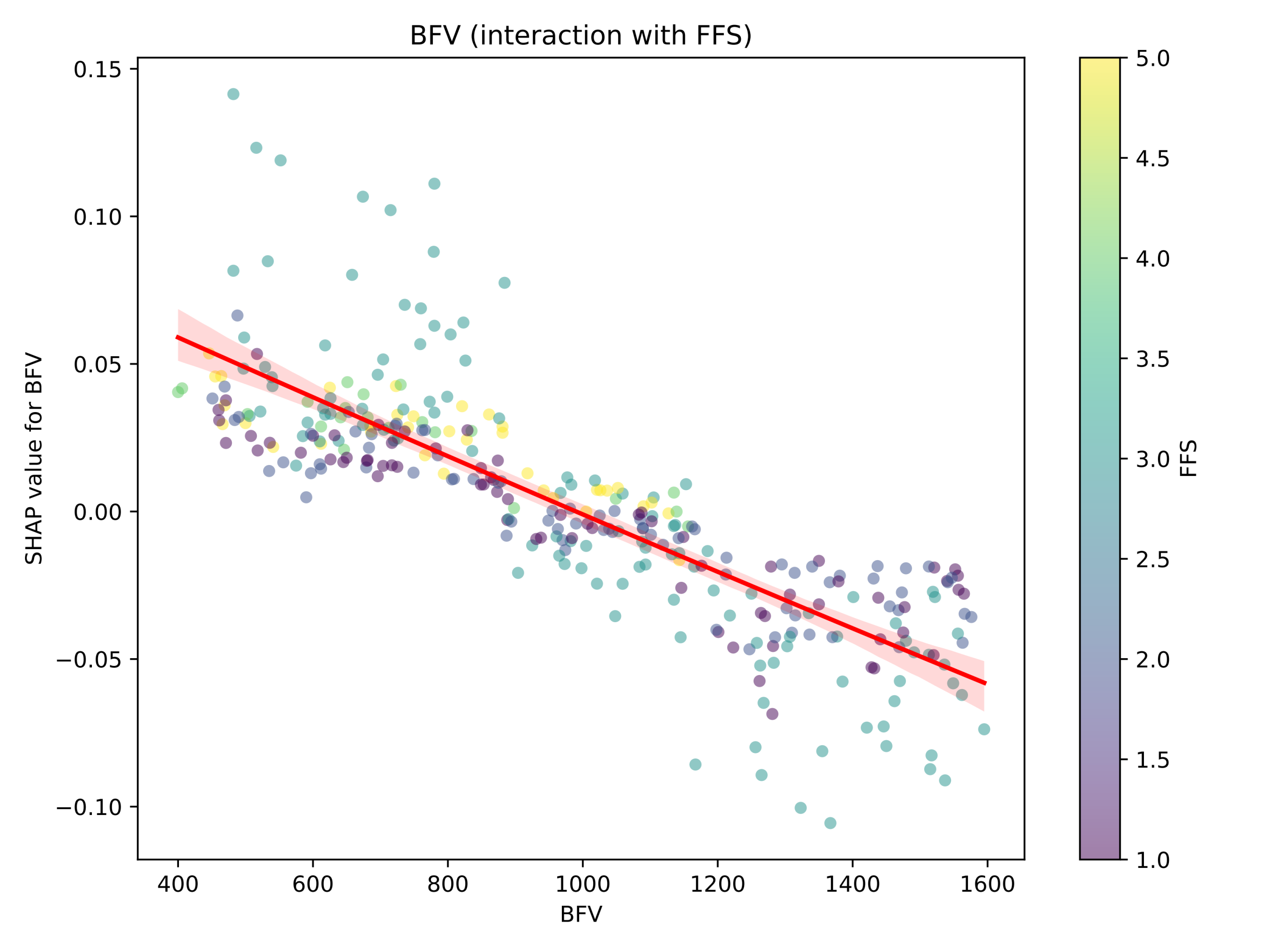 |
| --- | --- | --- | --- |
| A | B | C | D |
| 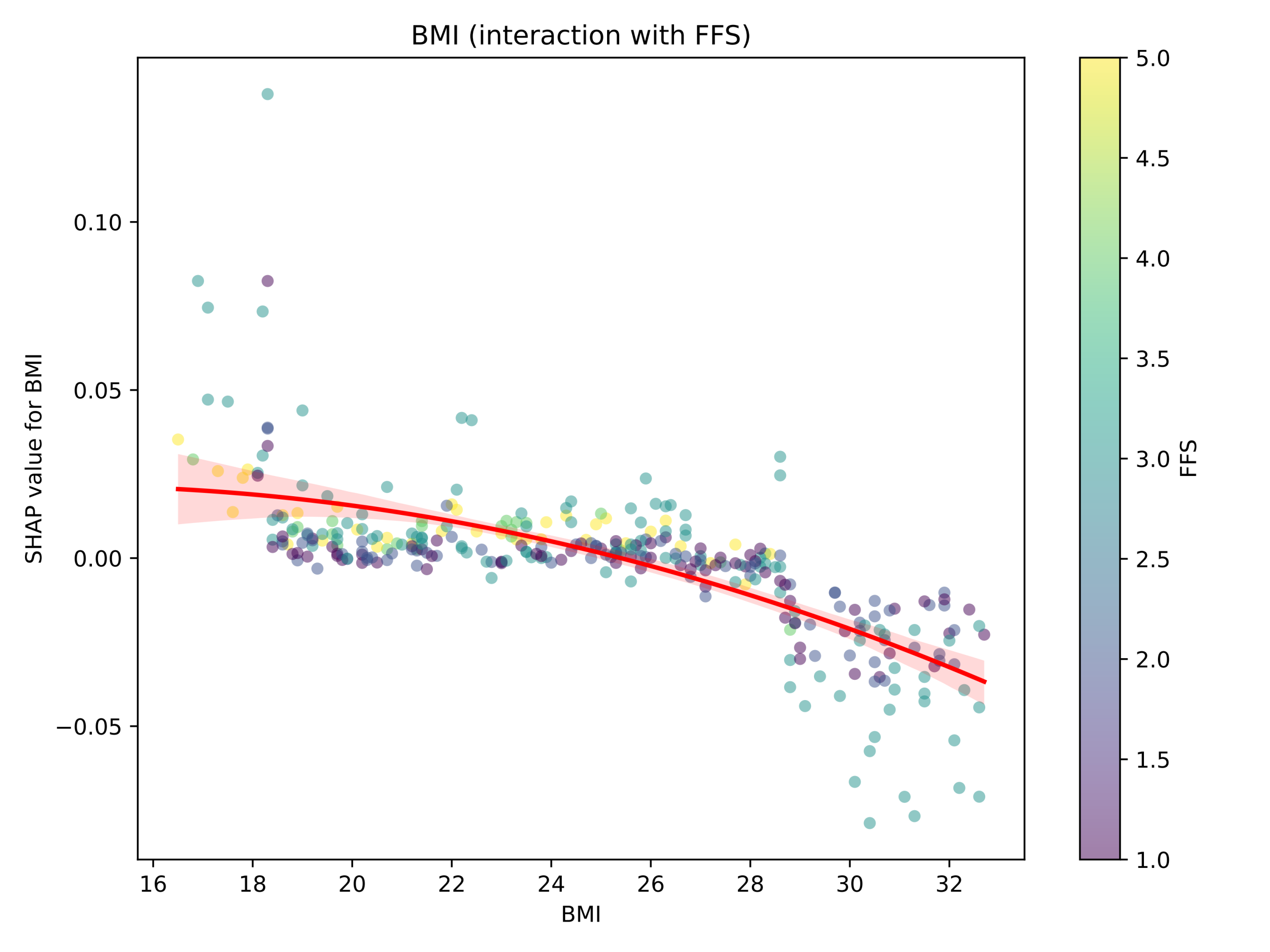 | 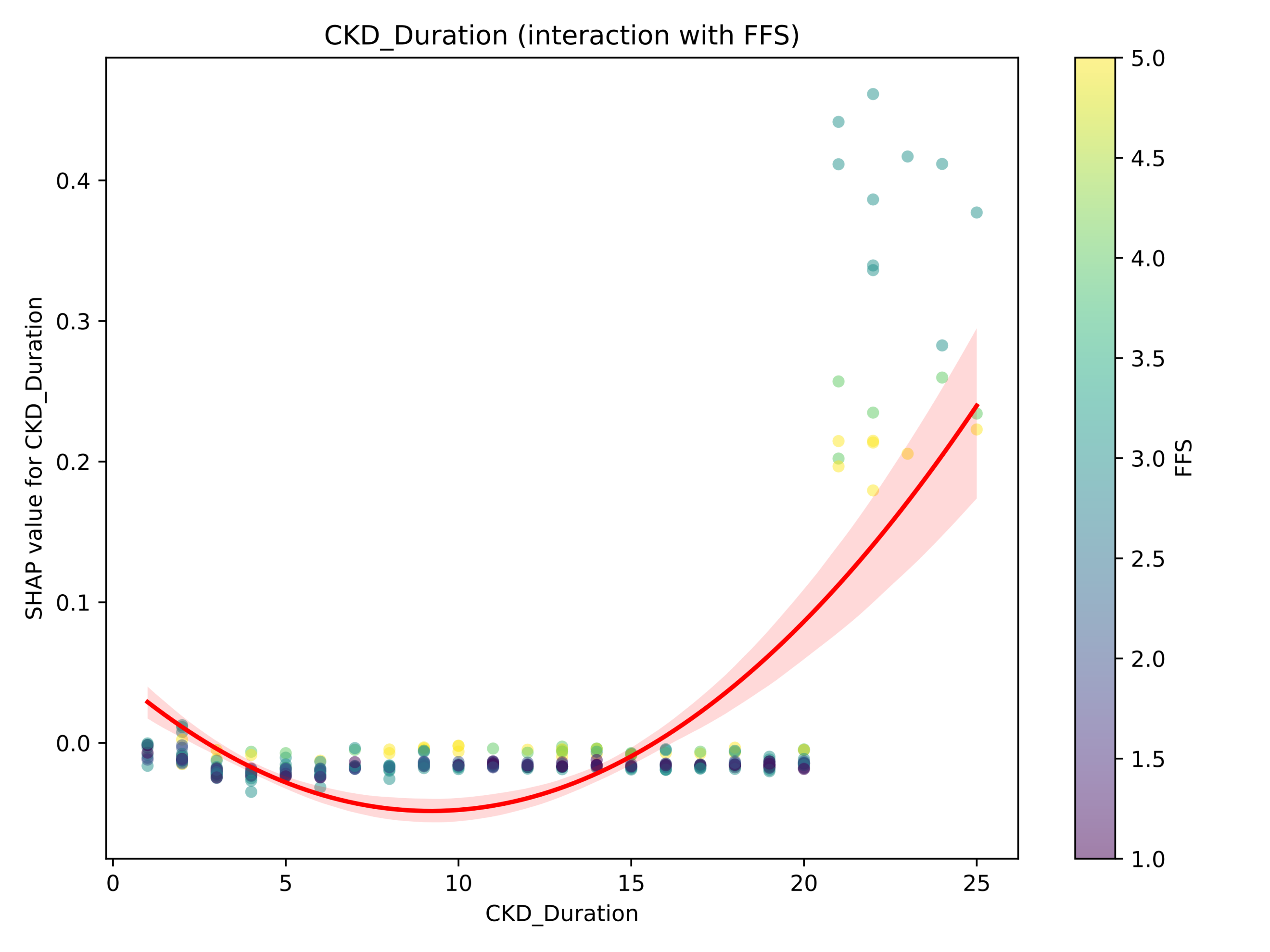 | 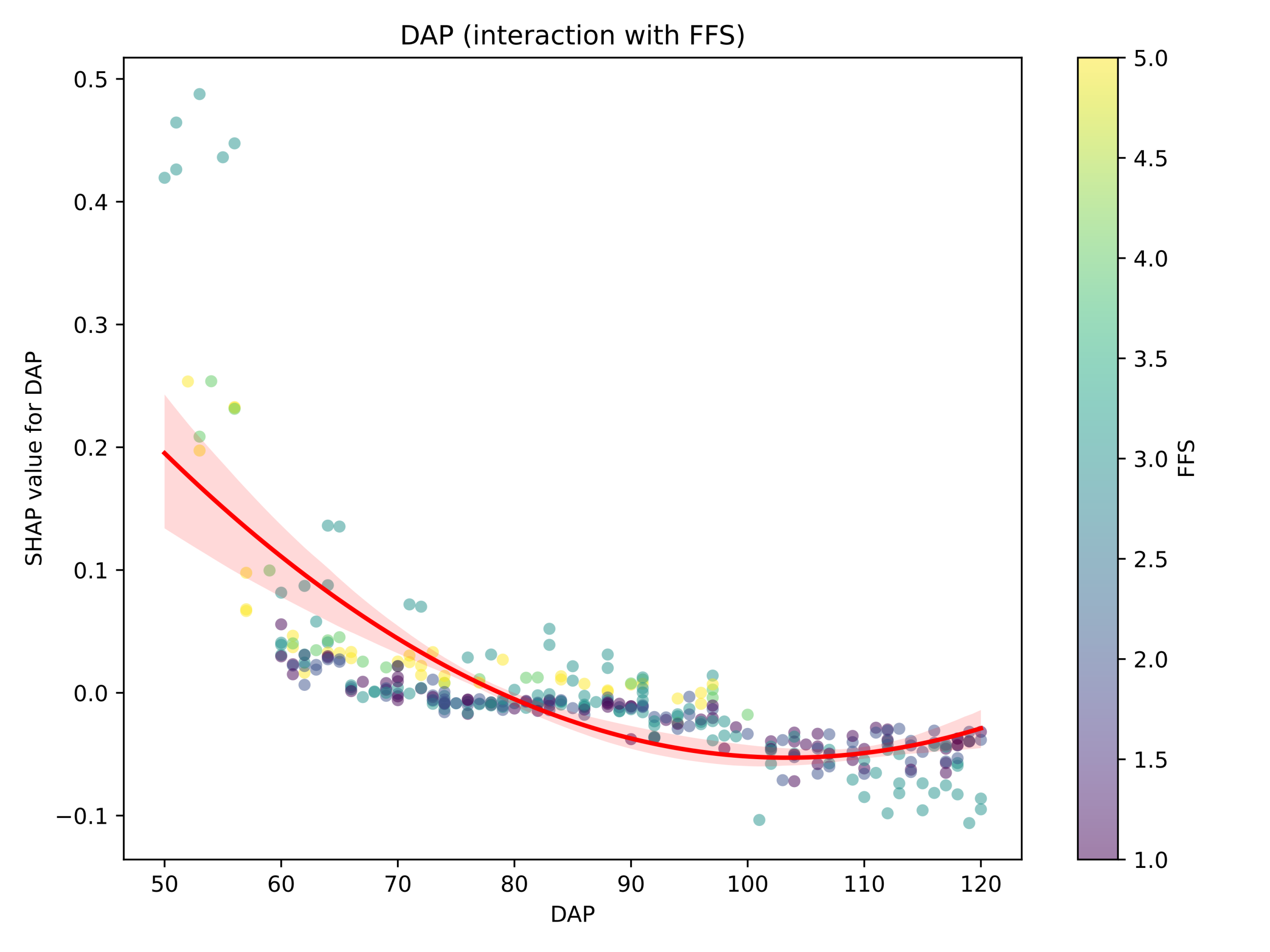 | 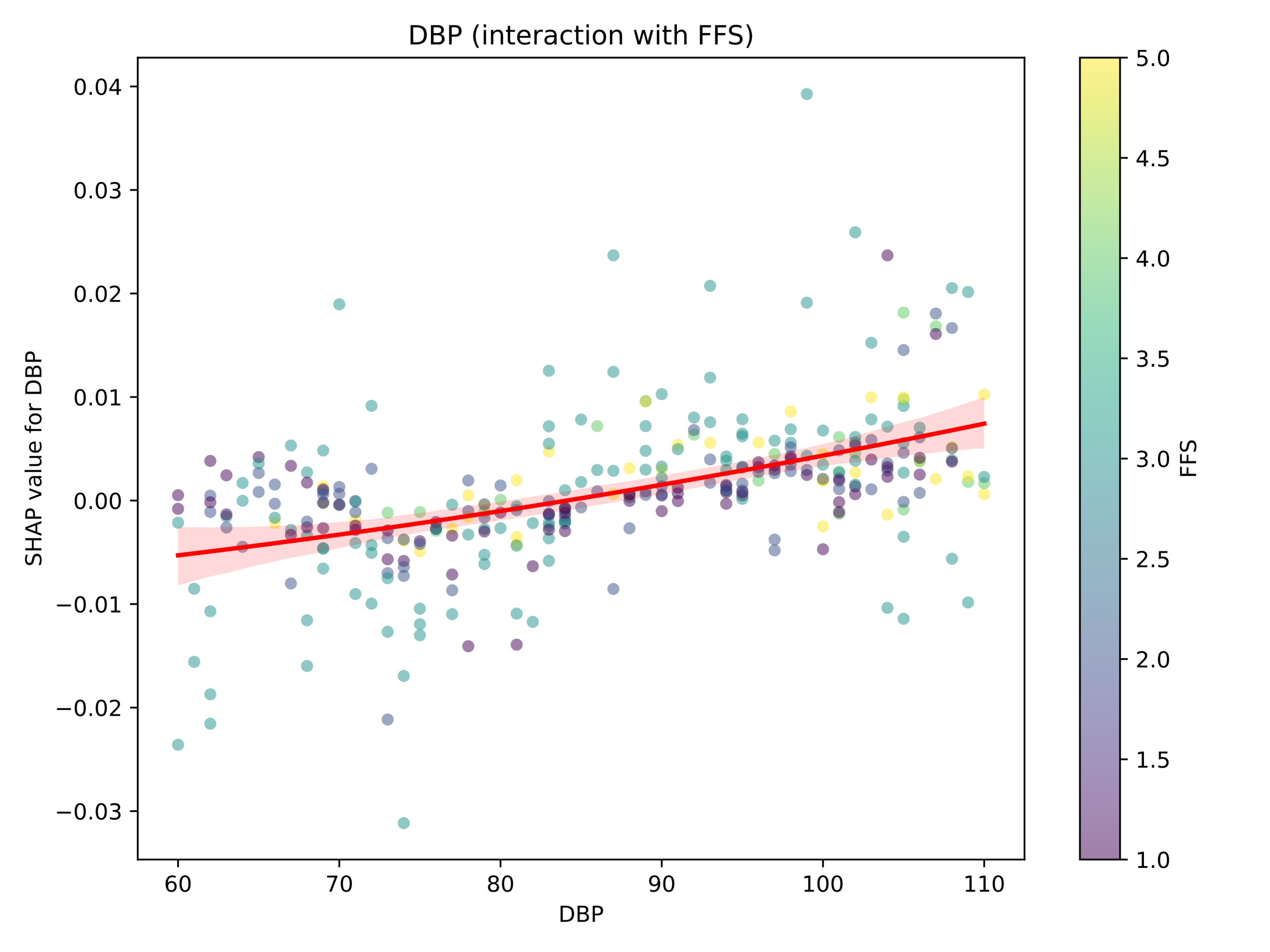 |
| E | F | G | H |
| 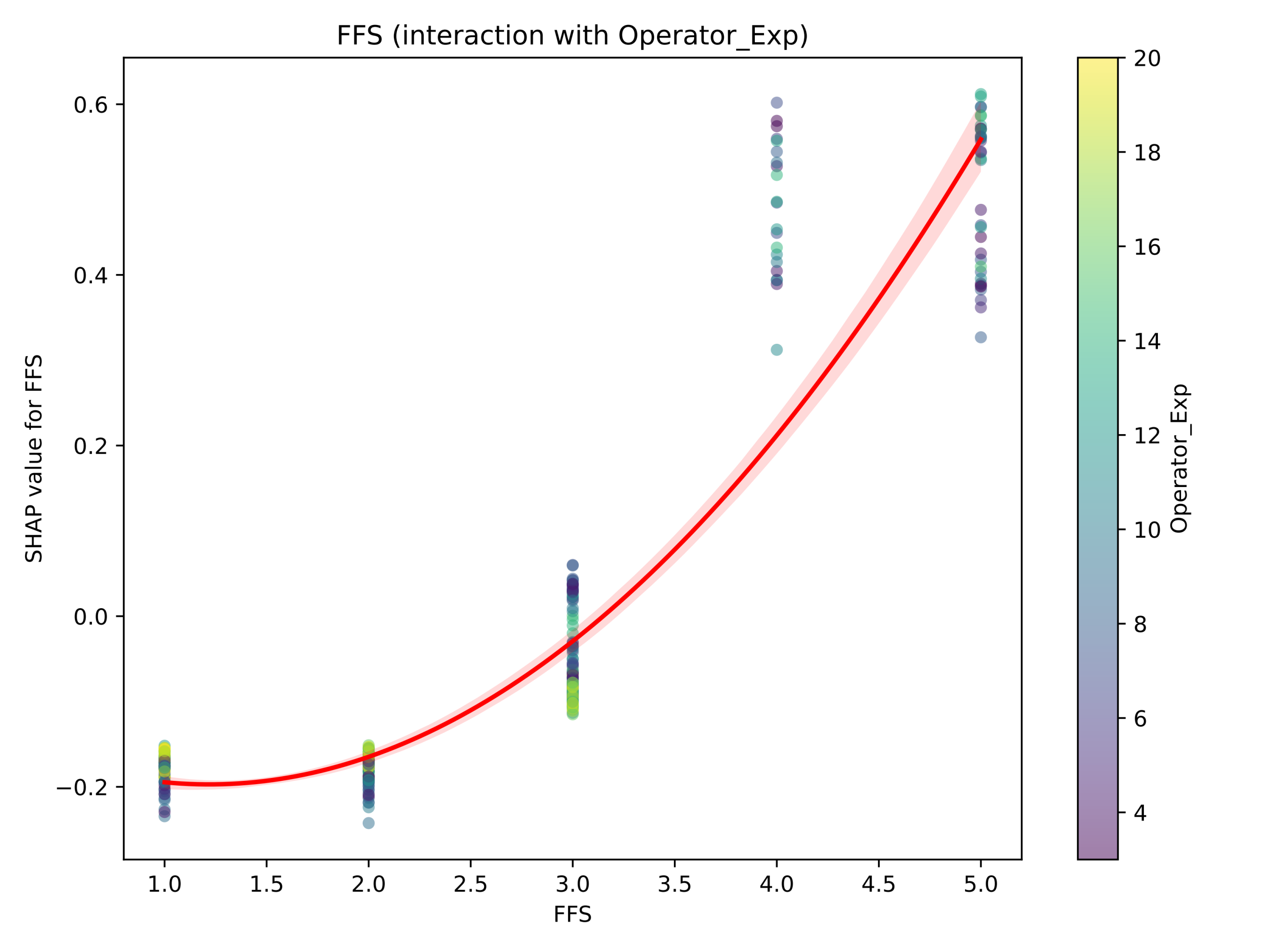 | 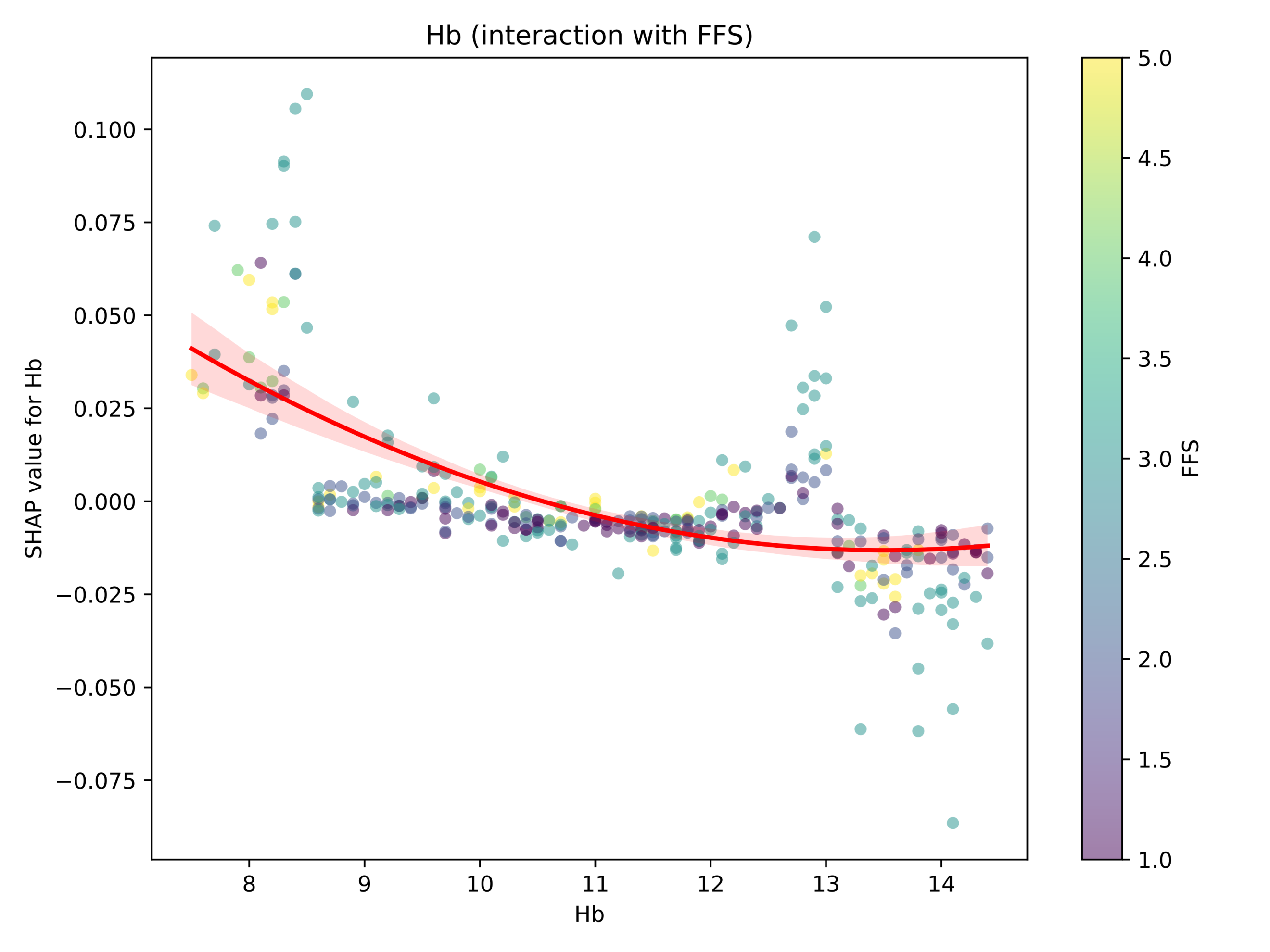 | 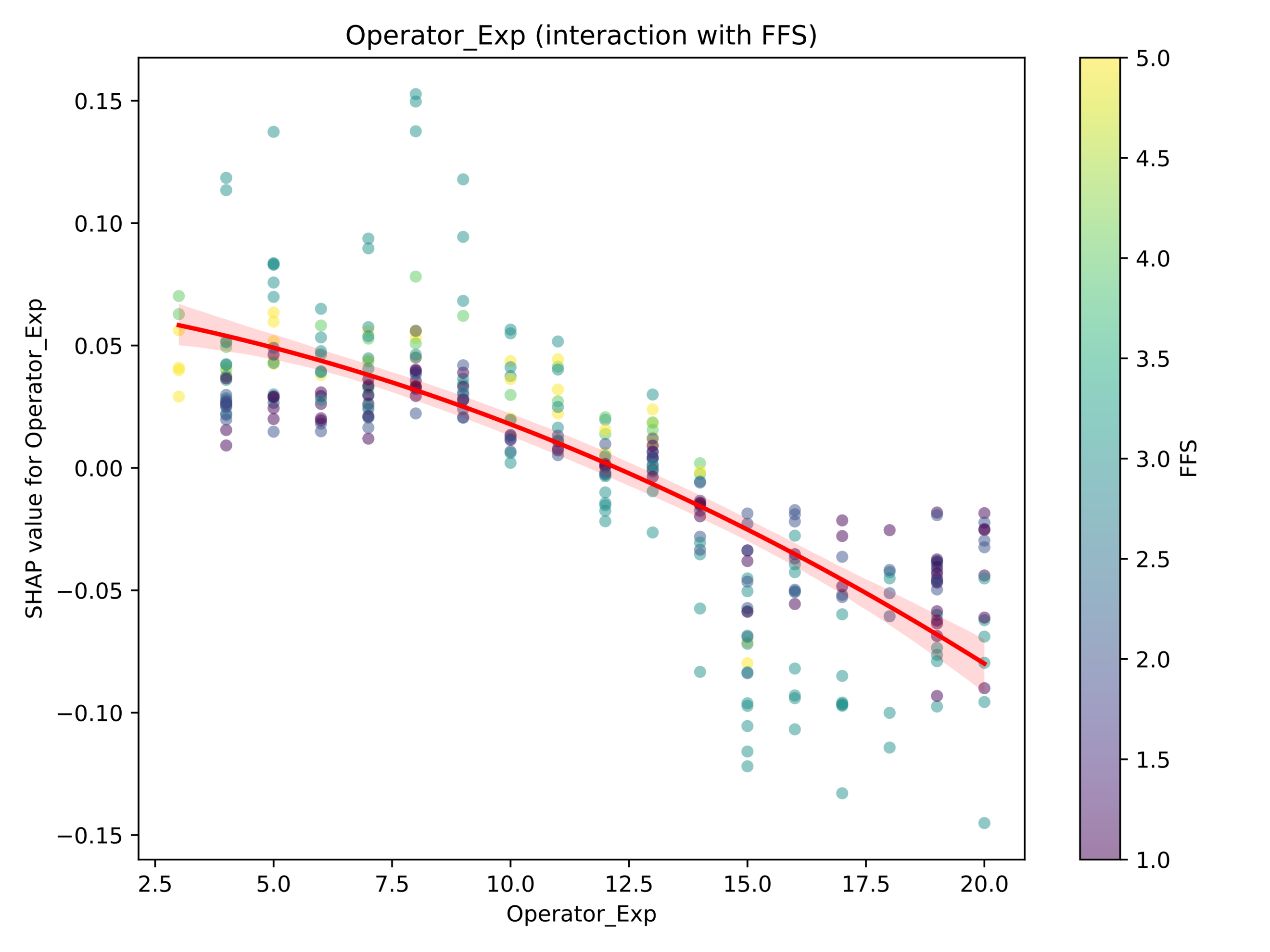 | 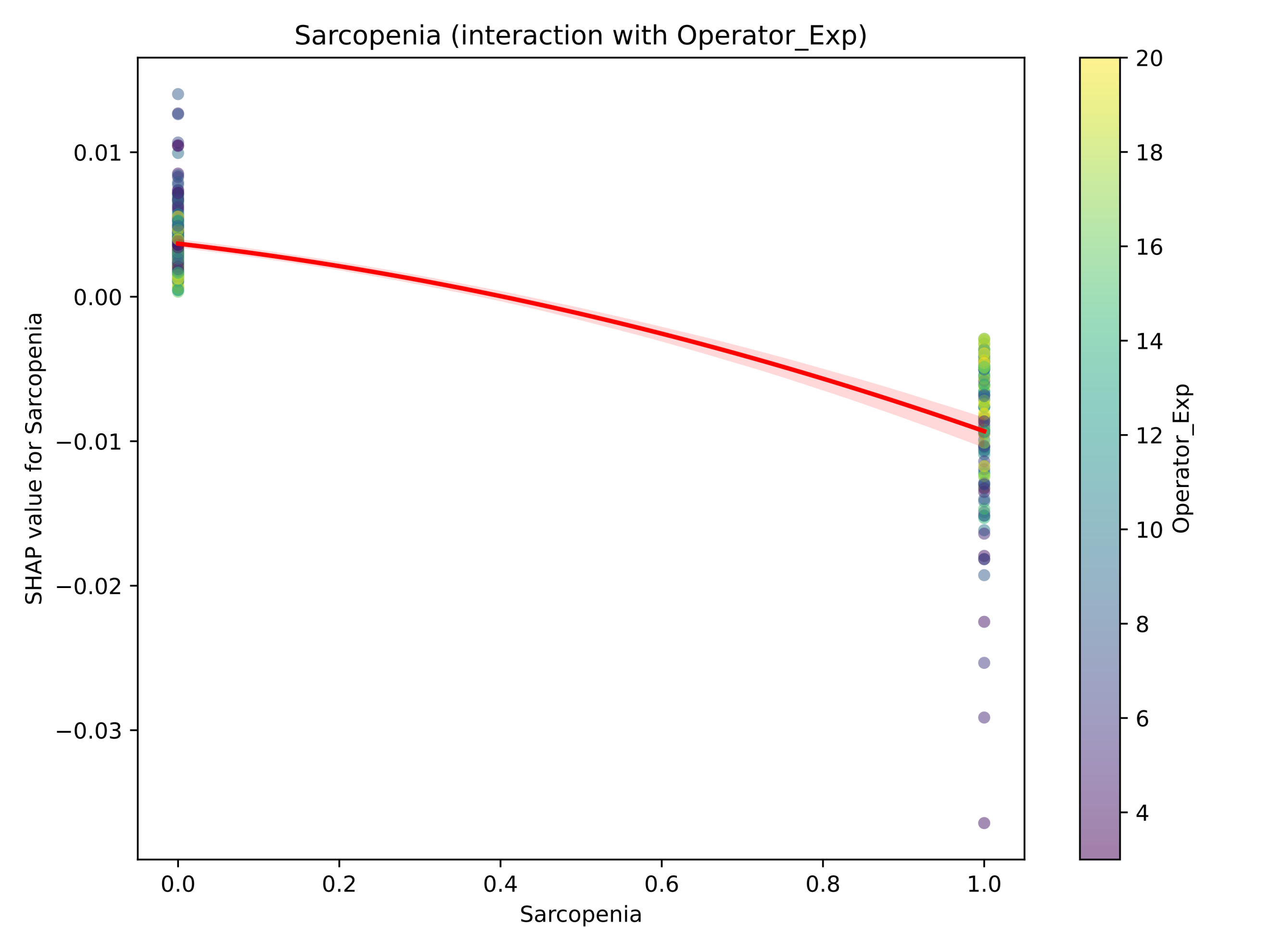 |
| I | J | K | L |
| 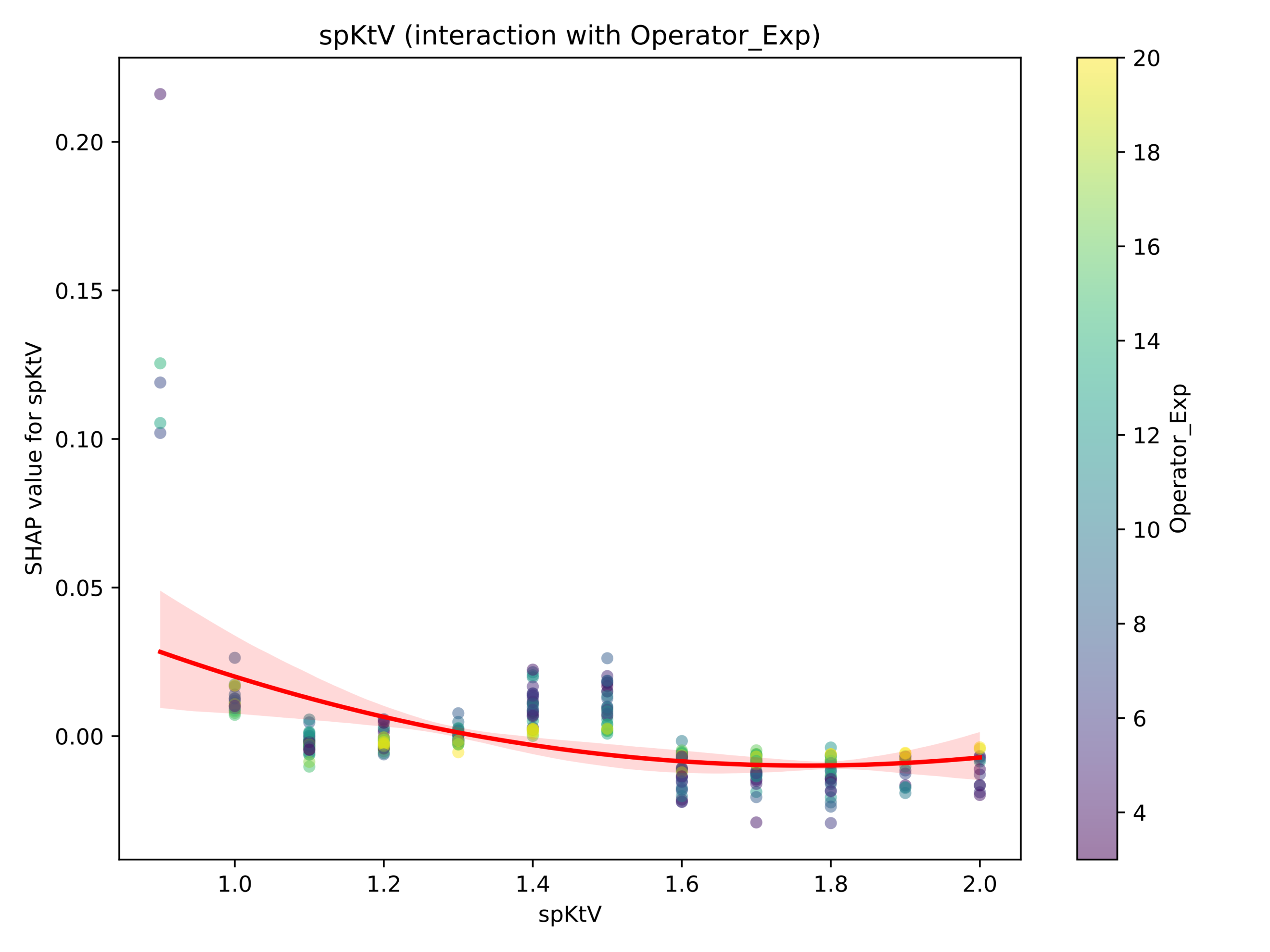 | 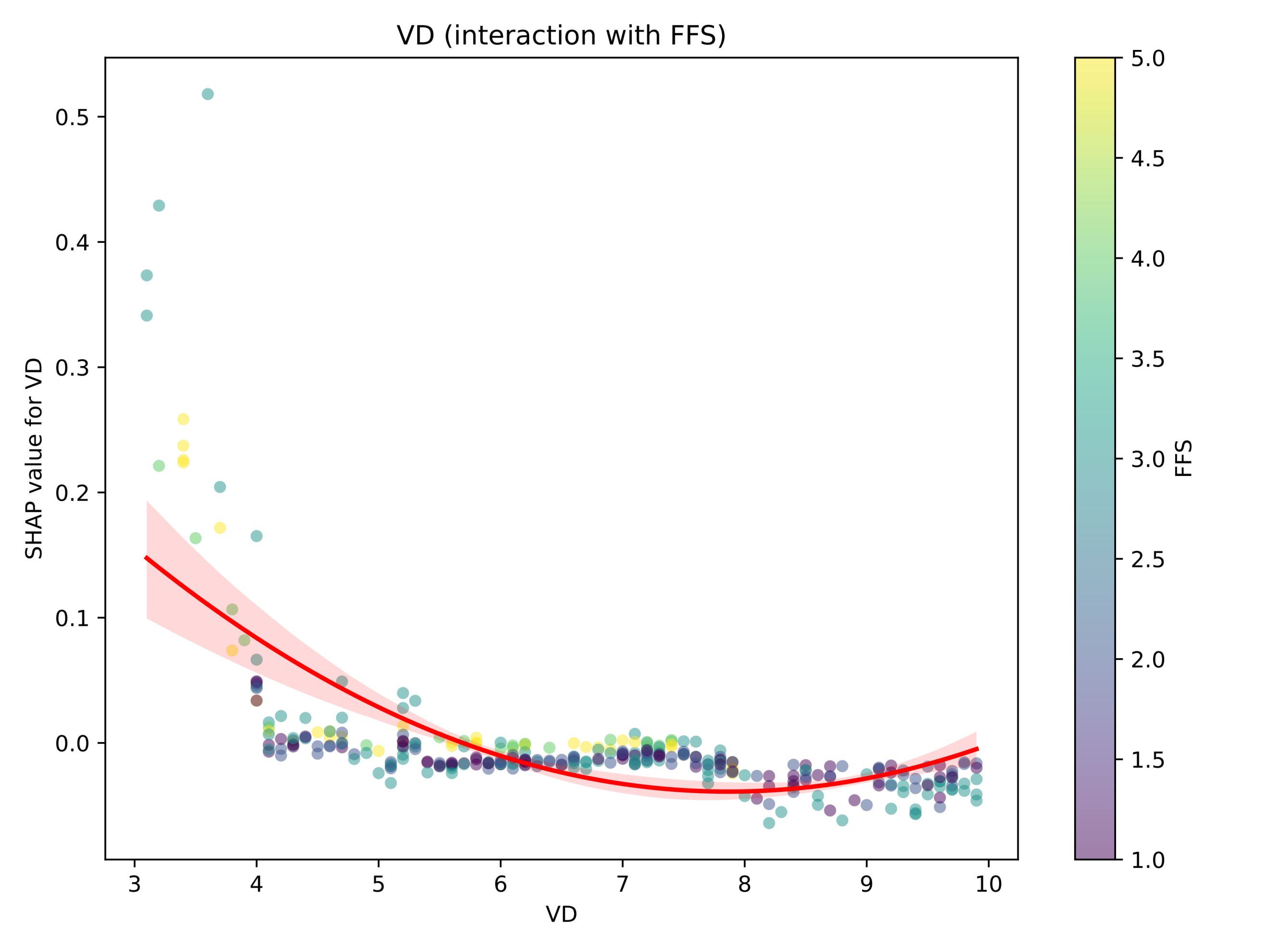 |  |  |
| M | N |  |  |

**Figure S1:** Partial Dependence Plots (PDP) illustrating the interaction effects of feature variables on DASS risk in ESRD patients.

**Note:**

A. Interaction between high ABI and low Fried frailty scores significantly reduces DASS risk.

B. The interaction between age and BFV shows a minimal synergistic effect on DASS prediction.

C. The interaction between ABI and DAP shows limited predictive value for DASS risk.

D. The combination of low BFV and high Fried frailty scores significantly increases DASS risk.

E. The interaction between low BMI and high Fried frailty scores markedly elevates the risk of DASS.

F. Longer CKD duration combined with high Fried frailty scores is associated with a significant increase in DASS risk.

G. Interaction between high GDP and low Fried frailty scores significantly reduces DASS risk.

H. High DBP combined with high Fried frailty scores contributes to increased DASS risk.

I. High Fried frailty scores in the context of limited operator experience significantly reduce DASS risk.

J. The interaction between low Hb and high Fried frailty scores increases the risk of DASS.

K. Extensive operator experience paired with low Fried frailty scores significantly reduces DASS risk.

L. The interaction between sarcopenia and operator experience shows limited predictive value for DASS.

M. The interaction between spKt/V and operator experience demonstrates a minimal synergistic effect on DASS risk.

N. High VD combined with low Fried frailty scores significantly reduces DASS risk.

**Table S1.** Missing Data Distribution and Handling Strategies (N=324)

| Variable | Missing, n | Missing, % | Handling Method |
| --- | --- | --- | --- |
| **Demographics** |  |  |  |
| Age | 0 | 0.00% | None |
| BMI | 12 | 3.70% | Multiple Imputation |
| **Clinical History** |  |  |  |
| CKD duration | 8 | 2.50% | Multiple Imputation |
| Diabetes mellitus | 0 | 0.00% | None |
| **Functional Indicators** |  |  |  |
| Fried Frailty Score (FFS) | 15 | 4.60% | Multiple Imputation |
| Sarcopenia (AWGS 2019) | 10 | 3.10% | Multiple Imputation |
| Hemodynamic Markers |  |  |  |
| Distal Arterial Pressure (DAP) | 18 | 5.60% | Multiple Imputation |
| Brachial artery diameter | 5 | 1.50% | Multiple Imputation |
| **Laboratory Results** |  |  |  |
| Albumin | 22 | 6.80% | Multiple Imputation |
| Hemoglobin | 7 | 2.20% | Multiple Imputation |

**Table S2:** Characteristics of Variables Included in the LASSO Regression Analysis

| NO | Variables | Description/Units |
| --- | --- | --- |
| 1 | Age | years |
| 2 | BMI | kg/m² |
| 3 | CKD Duration | years |
| 4 | Diabetic Nephropathy | Yes/No |
| 5 | Other Kidney Diseases | Yes/No |
| 6 | spKt/V | Single-pool Kt/V |
| 7 | Systolic BP | mmHg |
| 8 | Diastolic BP | mmHg |
| 9 | Hemoglobin | g/dL |
| 10 | Serum Albumin | g/L |
| 11 | Fried Criteria | Number of items met |
| 12 | Sarcopenia | N, % |
| 13 | Blood Flow Velocity | mL/min |
| 14 | Vessel Diameter | mm |
| 15 | ABI | Ankle-Brachial Index |
| 16 | DAP | mmHg |
| 17 | Operator Experience | years |

**Note:** The candidate features listed above were selected for the LASSO regression analysis based on their statistical significance (P < 0.05) in the preceding univariate analysis (**see Table 1**).

**Abbreviations:** BMI, body mass index; CKD, chronic kidney disease; BP, blood pressure; ABI, ankle-brachial index; DAP, dialysate arterial pressure; spKt/V, single-pool Kt/V.

**Table S3.** Sensitivity Analysis and Robustness Check of Multivariable Logistic Regression Models.

| Variable | MICE Imputed Dataset (N=324) | | | | Complete Case Analysis (n=286) | | | |
| --- | --- | --- | --- | --- | --- | --- | --- | --- |
|  | OR | 95% CI | P value | E-value | OR | 95% CI | P value | E-value |
| CKD Duration | 1.23 | 1.05–1.46 | 0.01 | 1.95 | 1.21 | 1.03–1.42 | 0.02 | 1.85 |
| Sarcopenia | 1.4 | 1.04–1.91 | 0.03 | 2.17 | 1.37 | 1.01–1.88 | 0.04 | 2.05 |
| DAP | 0.87 | 0.79–0.95 | <0.001 | 1.64 | 0.88 | 0.80–0.97 | <0.001 | 1.59 |
| FFS | 2.33 | 1.09–3.57 | 0.02 | 2.77 | 2.29 | 1.06–3.53 | 0.03 | 2.69 |
| BMI | 0.69 | 0.53–0.91 | 0.01 | 2 | 0.71 | 0.54–0.93 | 0.01 | 1.92 |
| Operator Experience | 0.55 | 0.45–0.67 | <0.001 | 1.32 | 0.56 | 0.46–0.68 | <0.001 | 1.28 |

**Note:** E-value represents the minimum strength of association an unmeasured confounder would need to have with both the exposure and outcome to fully explain away the observed association.

**Abbreviations:** MICE, Multiple Imputation by Chained Equations; CC, Complete Case; OR, Odds Ratio; CI, Confidence Interval; CKD, Chronic Kidney Disease; DAP, Distal Arterial Pressure; FFS, Fried Frailty Score.

**Table S4:** Performance Evaluation of the Predictive Model via 10-Fold Cross-Validation

| Fold | Training AUC | Testing AUC |
| --- | --- | --- |
| 1 | 0.93 | 0.975 |
| 2 | 0.939 | 0.875 |
| 3 | 0.933 | 0.918 |
| 4 | 0.941 | 0.849 |
| 5 | 0.932 | 0.946 |
| 6 | 0.933 | 0.956 |
| 7 | 0.932 | 0.949 |
| 8 | 0.927 | 1 |
| 9 | 0.932 | 0.973 |
| 10 | 0.945 | 0.817 |
| Mean ± SD | 0.934±0.005 | 0.926±0.059 |

**Note:** The table presents the Area Under the Curve (AUC) for both the training and testing sets across each fold of the 10-fold cross-validation process. Mean ± SD represents the average value and standard deviation of the 10 iterations. The consistent AUC values between the training and testing sets indicate that the model maintains high discriminative power and robust generalization performance.

**Abbreviations:** AUC, Area Under the Curve; SD, Standard Deviation.
